# Supplementary material for: A numerical evaluation of real-time workloads for ramp controller through optimization of multi-type feature combinations derived from eye tracker, respiratory, and fatigue patterns
Source: PLoS One. 2024 Nov 8;19(11):e0313565. doi: 10.1371/journal.pone.0313565 (PMC11548742; doi:10.1371/journal.pone.0313565)
Supplement: S1 Dataset — (PDF) [file pone.0313565.s001.pdf]

ID	Tester	Minute(s)	EAR_avg	PERCLOS_avg	M_avg	Mild_Max	Moderate_Max	Severe_Max	Breaths_Count	Blinks_Count	Aoi000_FixRate	Aoi001_FixRate	Aoi000-Aoi001_TransferCount	Aoi-Nowhere_TransferCount
1	Tester1	1	0.296409265	0.58227957	0.496518479	0	0	0	20	31	77.89%	0.07%	4	11
2	Tester1	2	0.288587843	0.495634409	0.433520439	0	0	0	18	27	76.12%	0.22%	6	8
3	Tester1	3	0.298968119	0.504344086	0.442731296	0	0	0	17	22	82.54%	0.00%	0	10
4	Tester1	4	0.300165151	0.46172043	0.413253846	0	0	0	20	24	98.76%	0.10%	3	10
5	Tester1	5	0.306515779	0.338516129	0.328916024	0	0	0	16	21	88.06%	0.27%	4	11
6	Tester1	6	0.302173952	0.283397849	0.28903068	0	0	0	16	20	90.32%	0.06%	6	14
7	Tester1	7	0.302508516	0.357698925	0.341141802	0	0	0	19	21	89.00%	0.06%	4	11
8	Tester1	8	0.282526227	0.518451613	0.447673997	0	0	0	16	18	88.48%	0.21%	2	7
9	Tester1	9	0.286275861	0.688387097	0.567753726	0	1	0	16	12	97.61%	0.09%	5	11
10	Tester1	10	0.277669832	0.699397849	0.572879444	0	2	0	17	17	87.49%	0.25%	5	14
11	Tester1	11	0.297539819	0.574967742	0.491739365	0	2	0	19	18	79.97%	0.31%	5	12
12	Tester1	12	0.298428192	0.349892473	0.334453189	0	2	0	13	16	92.94%	0.05%	2	10
13	Tester1	13	0.27948361	0.546064516	0.466090244	0	2	0	19	21	98.56%	0.05%	2	12
14	Tester1	14	0.289657566	0.607096774	0.511865012	0	2	0	15	19	87.58%	0.01%	1	10
15	Tester1	15	0.296758895	0.408752688	0.37515455	0	2	0	18	17	78.83%	0.00%	0	9
16	Tester1	16	0.289873051	0.43688172	0.39277912	0	2	0	18	17	75.71%	0.24%	3	7
17	Tester1	17	0.294607238	0.469032258	0.416704752	0	2	0	18	15	96.81%	0.13%	2	13
18	Tester1	18	0.302242649	0.418193548	0.383408279	0	2	0	15	14	98.15%	0.01%	1	13
19	Tester1	19	0.281524797	0.501096774	0.435225181	0	2	0	16	24	84.96%	0.00%	0	7
20	Tester1	20	0.281269178	0.723376344	0.590744194	3	2	0	16	21	88.92%	0.06%	4	13
21	Tester1	21	0.300476682	0.612344086	0.518783865	3	2	0	18	20	90.48%	0.00%	0	13
22	Tester1	22	0.284612366	0.445419355	0.397177258	3	2	0	14	24	89.46%	0.00%	0	8
23	Tester1	23	0.27116755	0.732150538	0.593855641	6	4	0	15	19	91.00%	0.00%	0	9
24	Tester1	24	0.273108171	0.792817204	0.636904494	6	4	0	17	18	89.16%	0.00%	0	8
25	Tester1	25	0.262972046	0.872021505	0.689306667	6	4	0	19	16	84.63%	0.37%	2	11
26	Tester1	26	0.263701283	0.95688172	0.748927589	6	4	0	15	19	89.40%	0.00%	0	9
27	Tester1	27	0.271989133	0.925247312	0.729269858	6	4	0	15	21	92.52%	0.43%	4	7
28	Tester1	28	0.287166391	0.68516129	0.56576282	6	4	1	14	18	77.07%	0.00%	0	7
29	Tester1	29	0.285626809	0.536731183	0.46139987	6	4	1	18	20	82.81%	0.48%	6	7
30	Tester1	30	0.297447742	0.477182796	0.42326228	6	4	1	15	21	87.90%	0.00%	0	14
31	Tester1	31	0.297994408	0.463870968	0.414108	6	4	1	18	19	89.32%	0.35%	5	12
32	Tester1	32	0.291804266	0.37288172	0.348558484	6	4	1	18	22	76.05%	0.00%	0	16
33	Tester1	33	0.278664166	0.581849462	0.490893873	6	4	1	19	21	74.11%	0.63%	6	13
34	Tester1	34	0.267561285	0.848365591	0.674124299	6	4	1	15	20	84.71%	0.77%	6	15
35	Tester1	35	0.274720029	0.706365591	0.576871923	6	4	2	17	23	77.52%	0.38%	3	12
36	Tester1	36	0.253319117	0.917182796	0.718023692	6	4	2	19	21	70.17%	0.91%	2	16
37	Tester1	37	0.256259273	0.929677419	0.727651975	6	4	2	20	22	72.89%	0.97%	2	16
38	Tester1	38	0.26291525	0.909634409	0.715618661	6	4	2	13	18	85.34%	0.93%	3	12
39	Tester1	39	0.257340843	0.912301075	0.715813006	6	4	2	19	24	86.57%	0.23%	3	15
40	Tester1	40	0.277019804	0.829247312	0.66357906	6	4	3	17	21	89.74%	0.00%	0	14
41	Tester1	41	0.271113909	0.77911828	0.626716968	7	4	3	17	16	87.35%	0.93%	2	16
42	Tester1	42	0.291707996	0.674623656	0.559748958	7	5	4	14	28	88.54%	0.68%	4	13
43	Tester1	43	0.299556764	0.334989247	0.324359502	7	5	4	14	17	78.25%	0.27%	5	15
44	Tester1	44	0.297398012	0.395419355	0.366012952	7	5	4	18	26	66.43%	0.00%	0	13
45	Tester1	45	0.296535659	0.429634409	0.389704784	7	5	4	12	86	75.48%	0.81%	6	14
46	Tester1	46	0.310370674	0.275806452	0.286175718	7	5	4	19	18	79.04%	0.43%	2	14
47	Tester1	47	0.304954859	0.256602151	0.271107963	7	5	4	18	25	69.31%	0.53%	5	13
48	Tester1	48	0.310666265	0.255483871	0.272038589	7	5	4	19	30	89.16%	0.00%	0	16
49	Tester1	49	0.312229834	0.187612903	0.224997982	7	5	4	17	41	66.36%	0.80%	3	12
50	Tester1	50	0.292666356	0.248086022	0.261460122	7	5	4	21	20	75.65%	0.72%	5	14
51	Tester1	51	0.293426432	0.551354839	0.473976317	7	5	4	17	25	77.80%	0.83%	2	15
52	Tester1	52	0.281020147	0.521784946	0.449555506	7	5	4	19	27	73.96%	0.59%	6	14
53	Tester1	53	0.28123527	0.76516129	0.619983484	8	5	5	16	27	87.44%	0.27%	3	15
54	Tester1	54	0.293456908	0.583139785	0.496234922	10	5	5	15	17	65.09%	0.85%	2	13
55	Tester1	55	0.286232567	0.498494624	0.434816007	10	5	5	18	15	69.98%	0.00%	0	12
56	Tester1	56	0.277976136	0.732	0.595792841	10	5	5	13	19	81.84%	0.28%	4	12
57	Tester1	57	0.292380311	0.576967742	0.491591513	10	5	6	14	16	70.26%	0.00%	0	14
58	Tester1	58	0.285756385	0.580236559	0.491892507	10	5	6	20	22	81.10%	0.55%	4	15
59	Tester1	59	0.254752577	0.841311828	0.665344053	10	5	6	19	30	85.26%	0.57%	6	12
60	Tester1	60	0.294943383	0.73683871	0.604270112	12	5	7	13	35	68.40%	0.23%	3	13
61	Tester1	61	0.285961267	0.539139785	0.46318623	12	5	7	20	31	61.54%	0.00%	0	19

62	Tester1	62	0.280132639	0.670258064	0.553220437	12	5	7	16	22	74.60%	1.37%	4	17
63	Tester1	63	0.294173517	0.481741935	0.42547141	12	5	7	18	22	67.47%	0.00%	0	23
64	Tester1	64	0.288720454	0.513591398	0.446130115	12	5	7	15	18	72.58%	1.69%	2	23
65	Tester1	65	0.267910641	0.758817204	0.611545235	12	6	7	22	34	71.49%	1.84%	2	17
66	Tester1	66	0.280751573	0.835956989	0.669395364	13	6	8	16	27	71.33%	1.29%	2	21
67	Tester1	67	0.27721063	0.628967742	0.523440608	13	6	8	18	36	68.43%	0.00%	0	21
68	Tester1	68	0.269116764	0.749462366	0.605358685	15	7	8	17	26	67.26%	1.03%	2	23
69	Tester1	69	0.279061659	0.813032258	0.652841078	15	7	8	20	26	70.94%	1.39%	2	22
70	Tester1	70	0.280514903	0.74083871	0.602741568	15	8	9	18	28	68.82%	0.00%	0	21
71	Tester1	71	0.262843808	0.687225806	0.559911207	15	8	9	15	25	68.48%	1.50%	5	18
72	Tester1	72	0.281776089	0.854709677	0.682829601	15	8	10	19	39	68.34%	0.00%	0	19
73	Tester1	73	0.292553155	0.517483871	0.450004656	15	8	10	17	24	65.89%	0.00%	0	22
74	Tester1	74	0.267350782	0.620129032	0.514295557	16	8	10	19	28	70.44%	0.00%	0	23
75	Tester1	75	0.27447388	0.797096774	0.640309906	16	8	11	19	21	70.83%	2.00%	5	17
76	Tester1	76	0.280219694	0.651634409	0.540209994	17	8	12	14	21	65.54%	1.03%	3	19
77	Tester1	77	0.280736602	0.746688172	0.606902701	18	10	12	17	27	65.25%	0.00%	0	17
78	Tester1	78	0.281889341	0.743225806	0.604824867	19	13	12	18	33	66.50%	0.00%	0	21
79	Tester1	79	0.290792595	0.512602151	0.446059284	19	13	12	15	23	61.83%	0.00%	0	16
80	Tester1	80	0.273553831	0.658043011	0.542696257	21	13	12	21	32	68.00%	1.88%	2	23
81	Tester1	81	0.279356262	0.697483871	0.572045588	22	14	12	17	24	65.94%	0.00%	0	23
82	Tester1	82	0.28652207	0.585913979	0.496096406	22	14	12	19	25	64.85%	1.77%	5	20
83	Tester1	83	0.280931792	0.609096774	0.510647279	22	14	12	17	36	66.41%	0.00%	0	21
84	Tester1	84	0.290195034	0.664666667	0.552325177	23	15	12	19	19	73.99%	1.19%	6	23
85	Tester1	85	0.284753914	0.618967742	0.518703593	23	15	12	18	29	68.84%	1.69%	2	19
86	Tester1	86	0.261705011	0.756430107	0.608012579	23	15	12	18	33	67.02%	0.00%	0	16
87	Tester1	87	0.261597398	0.85544086	0.677287822	23	15	12	15	46	68.57%	1.55%	6	23
88	Tester1	88	0.260063353	0.922946236	0.724081372	23	15	12	19	43	72.69%	1.14%	3	20
89	Tester1	89	0.247022089	0.93911828	0.731489422	23	15	12	20	30	63.79%	1.95%	6	21
90	Tester1	90	0.256832316	0.870817204	0.686621738	23	15	12	18	21	71.25%	1.87%	3	17
91	Tester2	1	0.290274003	0.576107527	0.49035747	0	0	0	18	23	79.90%	0.00%	0	7
92	Tester2	2	0.295140456	0.567827957	0.486021707	0	0	0	17	12	77.37%	0.59%	5	8
93	Tester2	3	0.290188518	0.486903226	0.427888813	0	0	0	20	16	78.46%	0.00%	0	7
94	Tester2	4	0.285802929	0.634967742	0.530218298	0	0	0	21	18	88.63%	0.00%	0	9
95	Tester2	5	0.267352684	0.827204301	0.659248816	0	0	0	18	22	88.29%	0.41%	6	7
96	Tester2	6	0.300457567	0.631075269	0.531889958	1	0	1	17	17	98.97%	0.32%	4	6
97	Tester2	7	0.291798953	0.457354839	0.407688073	1	0	1	21	20	87.41%	0.00%	0	8
98	Tester2	8	0.274179141	0.789569892	0.634952667	1	1	1	21	16	75.27%	0.84%	2	8
99	Tester2	9	0.280751768	0.802172043	0.645745961	1	2	2	21	23	78.02%	0.00%	0	9
100	Tester2	10	0.292246718	0.628967742	0.527951435	1	2	2	23	18	81.57%	0.00%	0	9
101	Tester2	11	0.282948272	0.57716129	0.488897385	1	2	2	20	16	87.57%	0.78%	6	6
102	Tester2	12	0.275871662	0.787827957	0.634241068	1	3	2	21	15	76.31%	0.90%	6	6
103	Tester2	13	0.296244102	0.660967742	0.55155065	4	3	3	16	20	85.60%	0.00%	0	9
104	Tester2	14	0.291931163	0.445462366	0.399403005	4	3	3	18	14	75.96%	0.96%	5	7
105	Tester2	15	0.305912777	0.399419355	0.371367382	4	3	3	21	9	88.12%	0.00%	0	7
106	Tester2	16	0.295947284	0.435935484	0.393939024	4	3	3	17	15	99.48%	0.00%	0	9
107	Tester2	17	0.296096431	0.51088172	0.446446134	4	3	3	20	19	77.83%	0.00%	0	6
108	Tester2	18	0.287995169	0.549462366	0.471022207	4	3	3	20	14	95.78%	0.00%	0	9
109	Tester2	19	0.294655133	0.562064516	0.481841701	4	3	3	20	20	81.56%	0.14%	5	6
110	Tester2	20	0.297631666	0.421763441	0.384523908	4	3	3	18	18	93.75%	0.86%	3	9
111	Tester2	21	0.295258818	0.457612903	0.408906678	4	3	3	19	23	79.94%	0.34%	6	7
112	Tester2	22	0.288000524	0.568666667	0.484466824	4	3	3	17	16	85.30%	0.24%	4	6
113	Tester2	23	0.291889214	0.614215054	0.517517302	4	3	3	18	15	85.18%	0.00%	0	8
114	Tester2	24	0.294609311	0.634021505	0.532197847	4	3	3	16	12	77.05%	0.53%	3	9
115	Tester2	25	0.291766049	0.609354839	0.514078202	4	3	3	20	19	80.90%	0.14%	4	7
116	Tester2	26	0.286855023	0.645505376	0.53791027	4	3	3	18	13	94.89%	0.22%	2	7
117	Tester2	27	0.302824808	0.493247312	0.436120561	4	3	3	21	16	77.75%	0.00%	0	7
118	Tester2	28	0.302373729	0.302451613	0.302428248	4	3	3	20	17	86.25%	0.00%	0	7
119	Tester2	29	0.292397806	0.456537634	0.407295686	4	3	3	16	19	95.87%	0.44%	6	9
120	Tester2	30	0.263744788	0.817247312	0.651196555	4	3	3	19	18	81.77%	0.00%	0	9
121	Tester2	31	0.26932968	0.858924731	0.682046216	4	3	3	20	18	77.89%	0.00%	0	11
122	Tester2	32	0.28736516	0.737397849	0.602388043	4	3	4	19	16	82.33%	0.00%	0	10
123	Tester2	33	0.313216659	0.448537634	0.407941342	4	3	4	17	19	87.41%	2.43%	6	10

124	Tester2	34	0.298579898	0.284967742	0.289051389	4	3	4	20	14	76.21%	1.54%	5	11
125	Tester2	35	0.29771971	0.431741935	0.391535268	4	3	4	19	20	72.54%	2.23%	6	12
126	Tester2	36	0.273671139	0.629634409	0.522845428	5	3	4	19	15	86.30%	0.00%	0	10
127	Tester2	37	0.256561392	0.911225806	0.714826482	5	3	4	17	17	71.00%	1.78%	4	10
128	Tester2	38	0.247639528	0.934473118	0.728423041	5	3	4	17	8	85.94%	0.00%	0	10
129	Tester2	39	0.256008873	0.948408602	0.740688683	5	3	4	17	25	86.27%	0.00%	0	13
130	Tester2	40	0.270259531	0.763268817	0.615366031	5	3	5	18	16	85.05%	1.67%	5	10
131	Tester2	41	0.264552528	0.844236559	0.67033135	5	3	5	18	19	85.44%	1.63%	3	11
132	Tester2	42	0.269505728	0.838043011	0.667481826	5	3	5	17	27	82.23%	2.81%	6	12
133	Tester2	43	0.238605689	0.83288172	0.654598911	9	3	6	19	15	88.69%	1.74%	4	13
134	Tester2	44	0.266355898	0.876645161	0.693558382	9	3	6	20	16	73.91%	2.60%	2	11
135	Tester2	45	0.264315021	0.843247312	0.669567625	9	3	6	19	25	78.87%	0.00%	0	12
136	Tester2	46	0.239941717	0.916817204	0.713754558	9	3	6	17	14	66.96%	1.69%	3	11
137	Tester2	47	0.243504026	0.962795699	0.747008197	9	3	6	21	20	73.75%	1.14%	5	11
138	Tester2	48	0.256516449	0.944602151	0.73817644	9	3	6	15	10	85.10%	0.00%	0	11
139	Tester2	49	0.252002663	0.863698925	0.680190046	9	3	6	16	15	65.61%	0.00%	0	10
140	Tester2	50	0.237104097	0.952795699	0.738088218	9	3	6	20	21	66.67%	2.67%	5	11
141	Tester2	51	0.239087832	0.974451613	0.753842479	9	3	6	17	21	83.50%	1.83%	5	12
142	Tester2	52	0.282350963	0.815569892	0.655604214	9	3	7	19	12	84.20%	0.00%	0	10
143	Tester2	53	0.305424001	0.543784946	0.472276663	9	3	7	19	15	83.84%	0.00%	0	12
144	Tester2	54	0.332178619	0.513827957	0.459333156	9	3	7	17	16	76.11%	1.65%	4	11
145	Tester2	55	0.311182525	0.388602151	0.365376263	9	3	7	18	14	72.25%	1.85%	4	12
146	Tester2	56	0.296216725	0.406924731	0.373712329	9	3	7	17	21	76.70%	2.41%	5	12
147	Tester2	57	0.276088919	0.616494624	0.514372912	10	3	7	19	13	84.34%	0.00%	0	12
148	Tester2	58	0.293166699	0.631913978	0.530289794	11	4	7	23	12	75.77%	0.00%	0	10
149	Tester2	59	0.300115066	0.438774194	0.397176455	11	4	7	22	13	76.24%	2.88%	2	10
150	Tester2	60	0.29978257	0.345655914	0.331893911	11	4	7	19	12	85.40%	0.00%	0	13
151	Tester2	61	0.302203304	0.371010753	0.350368518	11	4	7	19	15	63.27%	4.93%	4	15
152	Tester2	62	0.297287339	0.34572043	0.331190503	11	4	7	17	18	67.55%	0.00%	0	15
153	Tester2	63	0.297089831	0.403462366	0.371550605	11	4	7	16	5	71.74%	0.00%	0	15
154	Tester2	64	0.291198433	0.55888172	0.478576734	11	4	7	16	17	68.14%	0.00%	0	15
155	Tester2	65	0.293128845	0.464	0.412738654	11	4	7	19	19	74.31%	0.00%	0	18
156	Tester2	66	0.291742847	0.511784946	0.445772317	11	4	7	19	16	64.88%	0.00%	0	16
157	Tester2	67	0.289768088	0.549182796	0.471358383	11	4	7	22	18	69.38%	0.00%	0	18
158	Tester2	68	0.300690877	0.417354839	0.38235565	11	4	7	16	22	67.65%	0.00%	0	17
159	Tester2	69	0.294922163	0.399827957	0.368356219	11	4	7	20	16	69.56%	4.81%	6	17
160	Tester2	70	0.285701217	0.521053763	0.450447999	11	4	7	17	14	68.81%	0.00%	0	17
161	Tester2	71	0.288400132	0.617053763	0.518457674	11	4	7	17	18	63.85%	4.43%	3	15
162	Tester2	72	0.293430725	0.566924731	0.484876529	11	4	7	19	12	74.75%	4.22%	3	18
163	Tester2	73	0.289496894	0.550172043	0.471969498	11	4	7	20	19	60.19%	3.28%	3	17
164	Tester2	74	0.291964503	0.55916129	0.479002254	11	4	7	20	19	71.05%	4.12%	6	17
165	Tester2	75	0.296190038	0.576150538	0.492162388	11	4	7	17	20	61.49%	0.00%	0	15
166	Tester2	76	0.28965014	0.444602151	0.398116547	11	4	7	20	19	61.59%	0.00%	0	18
167	Tester2	77	0.287547305	0.554043011	0.474094299	11	4	7	17	18	62.14%	0.00%	0	16
168	Tester2	78	0.297169154	0.503419355	0.441544295	11	4	7	14	20	71.97%	0.00%	0	17
169	Tester2	79	0.294330769	0.460150538	0.410404607	11	4	7	18	20	66.01%	4.86%	6	15
170	Tester2	80	0.294247382	0.540623656	0.466710774	11	4	7	21	22	68.09%	3.23%	5	18
171	Tester2	81	0.296963748	0.427204301	0.388132135	11	4	7	21	23	61.04%	4.76%	2	15
172	Tester2	82	0.293206639	0.495612903	0.434891024	11	4	7	17	21	64.71%	0.00%	0	15
173	Tester2	83	0.295964416	0.469526882	0.417458142	11	4	7	22	18	65.69%	4.82%	6	15
174	Tester2	84	0.293532109	0.556	0.477259633	11	4	7	21	20	62.87%	4.35%	5	16
175	Tester2	85	0.277940108	0.703397849	0.575760527	13	5	7	17	18	64.57%	3.11%	5	18
176	Tester2	86	0.291500829	0.544451613	0.468566378	13	5	7	16	18	72.12%	0.00%	0	16
177	Tester2	87	0.291311875	0.569612903	0.486122595	13	5	7	16	27	66.82%	4.94%	6	17
178	Tester2	88	0.277877235	0.786516129	0.633924461	13	5	7	21	19	68.27%	0.00%	0	18
179	Tester2	89	0.287198102	0.748795699	0.61031642	16	6	8	19	22	61.74%	0.00%	0	16
180	Tester2	90	0.265800023	0.716946237	0.581602372	17	7	8	19	20	68.76%	0.00%	0	15
181	Tester3	1	0.285674619	0.476	0.418902386	0	0	0	9	31	83.11%	0.37%	3	12
182	Tester3	2	0.285820185	0.659849462	0.547640679	0	0	0	7	26	85.20%	0.44%	2	12
183	Tester3	3	0.291500984	0.500215054	0.437600833	0	0	0	9	29	78.75%	0.00%	0	13
184	Tester3	4	0.288973745	0.49911828	0.436074919	0	0	0	8	27	97.83%	0.00%	0	14
185	Tester3	5	0.295381315	0.508129032	0.444304717	0	0	0	8	17	90.47%	0.11%	6	8

186	Tester3	6	0.28039925	0.491483871	0.428158485	0	0	0	9	16	95.28%	0.00%	0	14
187	Tester3	7	0.269733318	0.735741935	0.59593935	2	0	0	9	24	89.92%	0.07%	5	11
188	Tester3	8	0.25180388	0.881655914	0.692700304	2	0	0	3	27	82.27%	0.16%	3	10
189	Tester3	9	0.279904905	0.777784946	0.628420934	2	0	1	7	23	86.14%	0.00%	0	8
190	Tester3	10	0.284787106	0.522989247	0.451528605	2	0	1	2	18	77.69%	0.00%	0	7
191	Tester3	11	0.276269547	0.682344086	0.560521724	2	0	1	5	16	77.39%	0.00%	0	10
192	Tester3	12	0.266732729	0.809397849	0.646598313	2	0	1	3	12	91.31%	0.04%	5	10
193	Tester3	13	0.279704979	0.706473118	0.578442677	4	0	2	3	15	76.02%	0.00%	0	10
194	Tester3	14	0.269426347	0.842752688	0.670754786	4	0	2	3	15	81.92%	0.00%	0	9
195	Tester3	15	0.270480397	0.872795699	0.692101108	4	0	2	4	13	84.02%	0.23%	3	10
196	Tester3	16	0.270745722	0.829698925	0.662012964	4	0	2	6	16	90.35%	0.03%	3	7
197	Tester3	17	0.268989748	0.866236559	0.687062516	4	0	2	4	11	77.30%	0.25%	2	10
198	Tester3	18	0.275786706	0.803655914	0.645295152	4	0	2	6	17	82.91%	0.03%	6	12
199	Tester3	19	0.269028238	0.814967742	0.651185891	4	0	2	5	18	76.85%	0.02%	3	11
200	Tester3	20	0.258217549	0.846967742	0.670342684	4	0	2	5	15	88.54%	0.00%	0	9
201	Tester3	21	0.264797737	0.822236559	0.655004913	4	1	3	7	14	84.39%	0.00%	0	8
202	Tester3	22	0.2614881	0.779096774	0.623814172	6	2	3	6	13	91.51%	0.00%	0	11
203	Tester3	23	0.259942402	0.848043011	0.671612828	6	2	3	7	10	89.12%	0.28%	4	10
204	Tester3	24	0.302005679	0.641075269	0.539354392	7	3	4	9	20	92.78%	0.49%	5	9
205	Tester3	25	0.301546709	0.358215054	0.34121455	7	3	4	5	15	78.93%	0.33%	2	7
206	Tester3	26	0.286681921	0.588473118	0.497935759	7	3	4	9	12	83.74%	0.26%	6	13
207	Tester3	27	0.306346805	0.484602151	0.431125547	7	3	4	5	13	82.71%	0.30%	5	13
208	Tester3	28	0.302752018	0.423204301	0.387068616	7	3	4	3	12	86.56%	0.23%	3	12
209	Tester3	29	0.302701076	0.370645161	0.350261936	7	3	4	4	22	98.19%	0.01%	1	9
210	Tester3	30	0.278291705	0.654817204	0.541859555	10	3	4	8	13	90.53%	0.14%	2	7
211	Tester3	31	0.300864336	0.514172043	0.450179731	10	3	4	7	12	82.84%	0.00%	0	13
212	Tester3	32	0.269049986	0.62888172	0.5209322	10	3	4	8	15	83.13%	0.00%	0	14
213	Tester3	33	0.270070421	0.847913978	0.674560911	10	3	4	4	18	87.89%	0.68%	2	14
214	Tester3	34	0.264324143	0.80655914	0.643888641	10	3	4	7	12	87.82%	0.51%	6	15
215	Tester3	35	0.265072707	0.865612903	0.685450844	10	3	4	7	19	69.32%	0.47%	3	13
216	Tester3	36	0.294893226	0.683849462	0.567162592	10	3	5	6	15	67.61%	0.00%	0	14
217	Tester3	37	0.285195235	0.63427957	0.529554269	10	3	5	8	11	70.48%	0.48%	3	12
218	Tester3	38	0.327715185	0.366580645	0.354921007	10	3	5	6	18	78.87%	0.32%	4	14
219	Tester3	39	0.310488151	0.280086022	0.28920666	10	3	5	7	12	71.00%	0.71%	3	16
220	Tester3	40	0.288147551	0.480172043	0.422564695	10	3	5	10	17	73.96%	0.88%	2	12
221	Tester3	41	0.263803368	0.754580645	0.607347462	10	3	5	11	14	74.51%	0.39%	4	14
222	Tester3	42	0.294675484	0.659612903	0.550131678	10	3	6	3	18	77.98%	0.00%	0	15
223	Tester3	43	0.283178224	0.696365591	0.572409381	11	3	6	9	19	66.99%	0.00%	0	16
224	Tester3	44	0.266610029	0.801419355	0.640976557	11	4	6	9	17	84.85%	0.93%	2	14
225	Tester3	45	0.264376574	0.847784946	0.672762434	11	4	6	9	12	67.16%	0.00%	0	12
226	Tester3	46	0.329862067	0.60683871	0.523745717	12	4	7	11	16	86.37%	0.22%	4	12
227	Tester3	47	0.345934865	0.12455914	0.190971857	12	4	7	4	16	82.04%	0.99%	3	15
228	Tester3	48	0.363179987	0.084688172	0.168235717	12	4	7	5	18	85.05%	0.83%	4	14
229	Tester3	49	0.35396972	0.044494624	0.137337153	12	4	7	5	15	89.08%	0.36%	4	15
230	Tester3	50	0.335802534	0.117978495	0.183325706	12	4	7	1	11	70.41%	0.72%	6	16
231	Tester3	51	0.312272981	0.226946237	0.25254426	12	4	7	4	23	86.29%	0.63%	5	12
232	Tester3	52	0.326353585	0.436774194	0.403648011	12	4	7	7	23	67.36%	0.79%	5	15
233	Tester3	53	0.324440565	0.360795699	0.349889159	12	4	7	6	15	76.92%	0.65%	2	16
234	Tester3	54	0.35177953	0.121870968	0.190843536	12	4	7	9	16	86.53%	0.00%	0	16
235	Tester3	55	0.344004081	0.133849462	0.196895848	12	4	7	5	15	76.57%	0.45%	4	14
236	Tester3	56	0.300686817	0.390731183	0.363717873	12	4	7	7	16	67.89%	0.28%	5	15
237	Tester3	57	0.289449171	0.680150538	0.562940128	12	4	7	5	15	77.57%	0.62%	4	15
238	Tester3	58	0.314305678	0.554021505	0.482106757	12	4	7	4	11	68.76%	0.21%	2	15
239	Tester3	59	0.289752063	0.561634409	0.480069705	12	4	7	6	17	69.73%	0.00%	0	13
240	Tester3	60	0.274564109	0.704989247	0.575861706	12	4	7	10	9	87.36%	0.91%	2	14
241	Tester3	61	0.277856124	0.776967742	0.627234257	12	4	8	7	17	64.31%	1.48%	2	22
242	Tester3	62	0.313487924	0.648709677	0.548143151	12	4	9	4	10	60.33%	1.18%	4	20
243	Tester3	63	0.279881896	0.514430108	0.444065644	12	4	9	9	10	66.97%	0.00%	0	23
244	Tester3	64	0.293289316	0.656473118	0.547517978	13	4	9	1	13	60.65%	0.00%	0	23
245	Tester3	65	0.287768523	0.597677419	0.50470475	13	4	9	8	12	62.41%	0.00%	0	18
246	Tester3	66	0.331735397	0.428860215	0.399722769	13	4	9	7	18	74.19%	1.15%	4	20
247	Tester3	67	0.329151758	0.177505376	0.222999291	13	4	9	9	17	65.57%	0.00%	0	21

248	Tester3	68	0.314478447	0.404494624	0.377489771	13	4	9	5	18	74.23%	1.49%	2	17
249	Tester3	69	0.332654302	0.242731183	0.269708119	13	4	9	5	15	60.75%	1.09%	4	19
250	Tester3	70	0.329179769	0.198236559	0.237519522	13	4	9	2	13	74.75%	1.25%	6	18
251	Tester3	71	0.287228682	0.467655914	0.413527745	13	4	9	2	16	71.59%	0.00%	0	17
252	Tester3	72	0.280510072	0.609526882	0.510821839	13	4	9	4	25	70.49%	1.80%	3	21
253	Tester3	73	0.304216688	0.685483871	0.571103716	15	4	9	4	17	64.93%	1.74%	5	22
254	Tester3	74	0.339381006	0.293225806	0.307072366	15	4	9	4	19	68.31%	0.00%	0	21
255	Tester3	75	0.316713367	0.35711828	0.344996806	15	4	9	8	21	67.25%	1.53%	6	23
256	Tester3	76	0.323450176	0.364150538	0.351940429	15	4	9	9	16	72.93%	1.27%	4	21
257	Tester3	77	0.343925041	0.16772043	0.220581813	15	4	9	7	17	70.66%	1.49%	5	18
258	Tester3	78	0.35175635	0.171784946	0.225776367	15	4	9	8	19	60.52%	1.47%	4	22
259	Tester3	79	0.357300997	0.094774194	0.173532235	15	4	9	7	23	62.81%	0.00%	0	19
260	Tester3	80	0.34048348	0.152021505	0.208560098	15	4	9	6	18	68.69%	0.00%	0	23
261	Tester3	81	0.327277215	0.222946237	0.25424553	15	4	9	4	19	68.79%	1.22%	5	19
262	Tester3	82	0.319516656	0.253978495	0.273639943	15	4	9	7	20	72.72%	1.43%	6	21
263	Tester3	83	0.310273233	0.289376344	0.295645411	15	4	9	10	18	65.58%	0.00%	0	21
264	Tester3	84	0.339471212	0.38372043	0.370445665	15	4	9	8	17	69.40%	1.14%	5	17
265	Tester3	85	0.311542673	0.219677419	0.247236995	15	4	9	11	17	72.64%	1.59%	3	16
266	Tester3	86	0.26405477	0.665053763	0.544754065	16	4	9	5	17	64.44%	1.09%	4	21
267	Tester3	87	0.260760852	0.856064516	0.677473417	16	4	9	6	18	71.48%	1.55%	3	19
268	Tester3	88	0.272163981	0.848494624	0.675595431	16	4	9	6	14	69.92%	1.66%	6	17
269	Tester3	89	0.270783864	0.721677419	0.586409353	17	4	10	7	5	60.03%	0.00%	0	16
270	Tester3	90	0.279341198	0.767806452	0.621266876	18	4	11	8	16	67.18%	0.00%	0	16
271	Tester4	1	0.318702299	0.282129032	0.293101012	0	0	0	14	37	80.35%	0.65%	2	6
272	Tester4	2	0.309632655	0.247483871	0.266128506	0	0	0	11	35	96.71%	0.50%	3	8
273	Tester4	3	0.308340045	0.236107527	0.257777282	0	0	0	11	36	89.13%	0.14%	4	6
274	Tester4	4	0.321783618	0.171225806	0.21639315	0	0	0	14	32	99.51%	0.00%	0	8
275	Tester4	5	0.312708539	0.17116129	0.213625465	0	0	0	12	32	93.26%	0.00%	0	7
276	Tester4	6	0.309081684	0.188129032	0.224414828	0	0	0	11	28	76.50%	0.45%	5	8
277	Tester4	7	0.308233895	0.243376344	0.262833609	0	0	0	11	38	95.27%	0.87%	6	8
278	Tester4	8	0.310712603	0.213569892	0.242712706	0	0	0	8	27	94.62%	0.00%	0	7
279	Tester4	9	0.281275104	0.461698925	0.407571778	0	0	0	8	30	92.75%	0.24%	6	6
280	Tester4	10	0.313563905	0.344688172	0.335350892	0	0	0	6	37	91.63%	0.00%	0	7
281	Tester4	11	0.263488411	0.604989247	0.502538996	0	0	0	4	36	89.05%	0.35%	4	9
282	Tester4	12	0.271824156	0.829419355	0.662140795	0	1	1	4	34	86.29%	0.24%	6	9
283	Tester4	13	0.303185264	0.459935484	0.412910418	0	1	1	10	34	98.69%	0.00%	0	9
284	Tester4	14	0.307989023	0.276107527	0.285671976	0	1	1	9	38	79.89%	0.92%	4	6
285	Tester4	15	0.307414946	0.277784946	0.286673946	0	1	1	5	28	76.55%	0.00%	0	6
286	Tester4	16	0.309000969	0.187225806	0.223758355	0	1	1	7	31	92.86%	0.80%	6	6
287	Tester4	17	0.312553423	0.153290323	0.201069253	0	1	1	6	30	95.34%	0.00%	0	6
288	Tester4	18	0.320290202	0.254365591	0.274142975	0	1	1	5	31	86.58%	0.83%	4	9
289	Tester4	19	0.302629097	0.171225806	0.210646794	0	1	1	4	28	86.97%	0.00%	0	6
290	Tester4	20	0.31163565	0.265634409	0.279434781	0	1	1	5	27	93.24%	0.61%	2	7
291	Tester4	21	0.31806299	0.196258065	0.232799542	0	1	1	2	26	98.77%	0.00%	0	8
292	Tester4	22	0.302040968	0.246193548	0.262947774	0	1	1	11	26	83.06%	0.23%	5	8
293	Tester4	23	0.296853983	0.287913978	0.29059598	0	1	1	7	28	77.97%	0.25%	4	9
294	Tester4	24	0.317894557	0.323763441	0.322002776	0	1	1	4	27	82.31%	0.00%	0	9
295	Tester4	25	0.314717371	0.196107527	0.23169048	0	1	1	4	31	92.90%	0.74%	3	7
296	Tester4	26	0.321566064	0.170408602	0.215755841	0	1	1	6	29	89.79%	0.26%	5	9
297	Tester4	27	0.302598579	0.261913978	0.274119359	0	1	1	8	32	91.05%	0.00%	0	7
298	Tester4	28	0.306770845	0.262666667	0.27589792	0	1	1	5	32	91.96%	0.06%	5	6
299	Tester4	29	0.309310213	0.297010753	0.300700591	0	1	1	4	31	78.95%	0.91%	4	6
300	Tester4	30	0.293579938	0.476537634	0.421650325	0	1	1	5	29	84.13%	0.52%	3	6
301	Tester4	31	0.284089271	0.428903226	0.385459039	0	1	1	5	24	85.81%	0.00%	0	11
302	Tester4	32	0.305069392	0.377849462	0.356015441	0	1	1	5	26	72.14%	2.92%	6	13
303	Tester4	33	0.311002078	0.290817204	0.296872666	0	1	1	5	30	70.11%	2.12%	3	10
304	Tester4	34	0.286721028	0.328451613	0.315932437	0	1	1	6	22	85.24%	0.00%	0	11
305	Tester4	35	0.302213877	0.448731183	0.404775991	0	1	1	7	30	73.30%	2.99%	6	12
306	Tester4	36	0.269213268	0.45688172	0.400581185	0	1	1	10	22	76.34%	1.88%	4	12
307	Tester4	37	0.286385316	0.59172043	0.500119896	0	1	1	5	24	89.14%	2.49%	3	13
308	Tester4	38	0.287042367	0.568752688	0.484239592	0	1	1	2	32	81.54%	0.00%	0	12
309	Tester4	39	0.283050296	0.58283871	0.492902185	0	1	1	8	32	81.87%	0.00%	0	12

310	Tester4	40	0.282307354	0.516473118	0.446223389	0	1	1	4	32	84.85%	1.76%	5	10
311	Tester4	41	0.298308543	0.471849462	0.419787186	0	1	1	7	27	89.35%	0.00%	0	10
312	Tester4	42	0.268875165	0.492494624	0.425408786	0	1	1	7	26	89.76%	2.73%	3	11
313	Tester4	43	0.271879986	0.649376344	0.536127437	0	1	1	6	31	70.70%	0.00%	0	13
314	Tester4	44	0.246030722	0.766	0.610009217	3	2	1	2	31	73.79%	0.00%	0	11
315	Tester4	45	0.255722774	0.791182796	0.630544789	4	2	2	4	24	77.95%	0.00%	0	12
316	Tester4	46	0.269773729	0.643397849	0.531310613	4	2	2	2	29	78.21%	2.02%	2	11
317	Tester4	47	0.273797063	0.60116129	0.502952022	4	2	2	12	26	67.07%	1.38%	3	10
318	Tester4	48	0.276719779	0.607655914	0.508375074	4	2	2	8	27	77.44%	0.00%	0	10
319	Tester4	49	0.279668084	0.717397849	0.58607892	7	2	2	7	30	70.38%	1.70%	6	12
320	Tester4	50	0.286483132	0.580537634	0.492321284	7	2	2	10	28	66.27%	0.00%	0	10
321	Tester4	51	0.267487805	0.51888172	0.443463546	7	2	2	12	26	85.40%	2.63%	4	10
322	Tester4	52	0.287765767	0.563849462	0.481024354	9	2	2	11	31	77.85%	0.00%	0	11
323	Tester4	53	0.293049148	0.445483871	0.399753454	9	2	2	4	27	69.94%	0.00%	0	13
324	Tester4	54	0.301991424	0.390344086	0.363838288	9	2	2	11	34	75.21%	1.06%	4	13
325	Tester4	55	0.294765434	0.405483871	0.37226834	9	2	2	5	32	70.81%	2.87%	5	11
326	Tester4	56	0.276965675	0.459655914	0.404848842	9	2	2	5	30	80.36%	0.00%	0	10
327	Tester4	57	0.299402314	0.52288172	0.455837899	9	2	2	6	35	70.64%	2.87%	3	12
328	Tester4	58	0.296969066	0.340322581	0.327316526	9	2	2	5	37	71.86%	1.26%	4	11
329	Tester4	59	0.289909806	0.437827957	0.393452512	9	2	2	7	33	76.32%	2.58%	3	10
330	Tester4	60	0.290684449	0.551698925	0.473394582	9	2	2	11	34	65.97%	0.00%	0	11
331	Tester4	61	0.295666446	0.515784946	0.449749396	9	2	2	11	30	69.60%	4.69%	3	18
332	Tester4	62	0.300511608	0.363913978	0.344893267	9	2	2	14	29	61.71%	4.81%	3	16
333	Tester4	63	0.299195571	0.409010753	0.376066198	9	2	2	7	26	71.96%	0.00%	0	18
334	Tester4	64	0.298504532	0.328172043	0.31927179	9	2	2	10	30	72.88%	3.52%	5	16
335	Tester4	65	0.284330014	0.468193548	0.413034488	9	2	2	9	29	73.50%	4.45%	4	17
336	Tester4	66	0.307367657	0.305655914	0.306169437	9	2	2	6	32	66.05%	0.00%	0	16
337	Tester4	67	0.265464225	0.536666667	0.455305934	9	2	2	10	30	66.10%	0.00%	0	18
338	Tester4	68	0.277460982	0.734516129	0.597399585	11	5	2	9	35	70.21%	4.83%	5	17
339	Tester4	69	0.263151414	0.698322581	0.567771231	12	6	2	10	33	61.26%	3.76%	2	17
340	Tester4	70	0.273448847	0.697333333	0.570167988	12	7	2	7	34	69.22%	4.09%	6	18
341	Tester4	71	0.275678557	0.64172043	0.531907868	12	7	2	2	35	74.12%	3.76%	5	16
342	Tester4	72	0.253847018	0.723849462	0.582848729	12	8	2	9	28	68.29%	4.21%	2	18
343	Tester4	73	0.284111792	0.749526882	0.609902355	13	8	3	9	38	74.99%	3.53%	2	15
344	Tester4	74	0.309287315	0.407096774	0.377753936	13	8	3	11	49	68.20%	0.00%	0	15
345	Tester4	75	0.261147288	0.453935484	0.396099025	13	8	3	16	33	63.75%	4.41%	5	18
346	Tester4	76	0.269978457	0.617354839	0.513141924	13	8	3	10	23	65.57%	3.34%	4	17
347	Tester4	77	0.286499041	0.596752688	0.503676594	13	8	3	10	35	72.30%	0.00%	0	15
348	Tester4	78	0.275691941	0.536430108	0.458208658	13	8	3	13	32	65.52%	0.00%	0	16
349	Tester4	79	0.282775115	0.558967742	0.476109954	13	8	3	10	29	71.86%	4.03%	5	17
350	Tester4	80	0.261372367	0.678795699	0.553568699	14	8	3	10	29	66.34%	0.00%	0	17
351	Tester4	81	0.250107643	0.818688172	0.648114013	14	8	3	10	31	70.87%	3.29%	4	16
352	Tester4	82	0.293120187	0.630989247	0.529628529	14	8	4	12	25	61.66%	3.15%	2	17
353	Tester4	83	0.296818935	0.408967742	0.3753231	14	8	4	14	32	70.02%	3.44%	6	18
354	Tester4	84	0.293807218	0.464666667	0.413408832	14	8	4	12	36	71.32%	3.64%	6	18
355	Tester4	85	0.288146516	0.507569892	0.441742879	14	8	4	11	33	69.18%	3.70%	3	15
356	Tester4	86	0.303240436	0.486645161	0.431623744	14	8	4	10	31	71.84%	3.86%	4	17
357	Tester4	87	0.298546463	0.254387097	0.267634907	14	8	4	10	37	73.60%	4.46%	6	17
358	Tester4	88	0.283514271	0.528623656	0.45509084	14	8	4	13	35	70.27%	4.67%	3	15
359	Tester4	89	0.294861769	0.594172043	0.504378961	14	8	4	14	35	69.16%	4.48%	2	17
360	Tester4	90	0.274832545	0.560193548	0.474585247	14	8	4	12	34	74.29%	3.80%	5	16
361	Tester5	1	0.291373617	0.514073708	0.44217394	0	1	0	13	23	88.67%	0.00%	3	8
362	Tester5	2	0.283300783	0.515328484	0.45050312	0	2	0	15	23	90.42%	0.00%	3	7
363	Tester5	3	0.28736411	0.525116541	0.450350148	1	3	0	15	23	89.18%	0.52%	2	9
364	Tester5	4	0.293280728	0.526047899	0.45370048	0	0	1	13	23	90.38%	1.13%	2	8
365	Tester5	5	0.285386919	0.527733886	0.456646046	1	1	1	13	23	89.93%	0.00%	1	8
366	Tester5	6	0.290694515	0.524695616	0.459768771	0	1	0	13	21	88.67%	1.80%	3	8
367	Tester5	7	0.294424277	0.522017103	0.460125222	1	2	0	13	22	86.81%	0.00%	2	7
368	Tester5	8	0.298115756	0.53881598	0.456201343	0	1	0	14	23	87.39%	0.00%	5	8
369	Tester5	9	0.291411383	0.524120927	0.46858361	2	0	0	13	23	85.79%	0.18%	3	10
370	Tester5	10	0.289729077	0.531299144	0.456306264	1	0	0	13	22	85.89%	1.49%	3	7
371	Tester5	11	0.290590946	0.534017991	0.461820661	0	3	1	11	22	86.10%	0.00%	2	10

372	Tester5	12	0.293069957	0.527251525	0.46031401	0	1	1	12	23	86.94%	0.00%	4	8
373	Tester5	13	0.291347674	0.535124789	0.454847527	1	2	0	13	20	86.51%	1.34%	1	8
374	Tester5	14	0.292259725	0.532613307	0.458709516	3	1	2	12	22	84.64%	0.00%	4	8
375	Tester5	15	0.290135121	0.54113955	0.456049641	2	1	0	12	22	86.72%	0.00%	2	9
376	Tester5	16	0.295001393	0.539902584	0.476186503	2	0	2	12	21	85.58%	0.66%	2	10
377	Tester5	17	0.290732743	0.540799111	0.46457318	3	1	1	11	20	85.56%	0.00%	3	11
378	Tester5	18	0.288392135	0.549287983	0.464838161	3	1	3	13	22	84.88%	1.22%	2	10
379	Tester5	19	0.286532281	0.535167671	0.461259714	3	2	1	14	21	84.08%	0.46%	2	10
380	Tester5	20	0.289273231	0.537060788	0.467402129	2	0	4	12	22	85.08%	0.43%	1	10
381	Tester5	21	0.29285519	0.549717798	0.46274976	3	1	1	12	22	85.09%	0.00%	2	11
382	Tester5	22	0.288382454	0.552480279	0.472672635	3	2	1	12	21	84.21%	0.75%	4	11
383	Tester5	23	0.290455129	0.552525864	0.469590742	2	2	2	13	22	82.70%	1.60%	1	11
384	Tester5	24	0.292242585	0.554484608	0.473318316	4	1	2	12	20	85.32%	0.33%	1	8
385	Tester5	25	0.28571391	0.543790267	0.478469025	3	2	1	13	20	84.23%	1.89%	2	10
386	Tester5	26	0.289041952	0.552193332	0.469625418	4	1	3	13	23	82.85%	0.00%	2	10
387	Tester5	27	0.285680046	0.549281538	0.469366221	5	2	4	13	21	84.00%	1.47%	2	10
388	Tester5	28	0.286695505	0.551781333	0.476030593	5	1	2	10	20	83.35%	0.00%	3	11
389	Tester5	29	0.294247275	0.547257265	0.476542042	5	3	3	10	20	82.52%	0.00%	3	10
390	Tester5	30	0.29818871	0.559551255	0.473994532	5	2	1	11	19	82.02%	0.00%	2	10
391	Tester5	31	0.283400169	0.559395319	0.473455133	5	3	3	11	21	81.47%	0.00%	3	11
392	Tester5	32	0.297474117	0.554166972	0.476528025	6	3	2	13	21	81.59%	1.59%	2	11
393	Tester5	33	0.284798037	0.563191151	0.489416187	6	1	2	11	20	81.22%	1.50%	3	11
394	Tester5	34	0.302298747	0.553337686	0.475070959	6	2	2	10	20	80.26%	2.49%	1	13
395	Tester5	35	0.291552422	0.562097691	0.477687501	5	3	3	13	21	80.59%	0.00%	2	11
396	Tester5	36	0.28847648	0.551823799	0.483995371	6	3	2	12	22	80.42%	1.15%	3	12
397	Tester5	37	0.285351692	0.557736875	0.474003645	5	3	3	12	21	78.75%	0.72%	3	11
398	Tester5	38	0.285838828	0.558133246	0.47006108	5	2	4	13	21	81.74%	1.12%	3	13
399	Tester5	39	0.285485359	0.566135105	0.477942325	8	2	3	12	20	79.96%	1.28%	3	13
400	Tester5	40	0.283258334	0.556003624	0.482005468	7	4	4	12	20	78.94%	1.64%	3	12
401	Tester5	41	0.281814901	0.564715125	0.47972182	8	4	3	12	22	76.61%	0.98%	2	13
402	Tester5	42	0.291911559	0.563937961	0.481722676	6	4	3	13	20	77.18%	0.04%	4	15
403	Tester5	43	0.287492857	0.562679621	0.471843144	7	4	5	12	21	76.94%	0.00%	4	13
404	Tester5	44	0.291403982	0.559756686	0.482892329	7	3	5	12	20	77.99%	0.57%	3	13
405	Tester5	45	0.287111289	0.561858543	0.476140915	10	5	4	13	22	78.55%	0.00%	4	13
406	Tester5	46	0.290978006	0.571348062	0.479844642	9	4	5	13	22	76.55%	2.31%	2	14
407	Tester5	47	0.29742956	0.559772747	0.474440177	9	3	4	11	20	76.95%	0.69%	3	14
408	Tester5	48	0.281980865	0.572889623	0.480194045	8	4	4	13	20	77.79%	1.93%	3	15
409	Tester5	49	0.28633013	0.565110704	0.487588837	9	3	4	13	20	76.32%	0.00%	2	14
410	Tester5	50	0.292545735	0.576819329	0.488175104	7	3	5	11	22	76.32%	0.74%	1	14
411	Tester5	51	0.289441055	0.560257009	0.484956661	7	3	5	11	22	76.24%	0.78%	2	12
412	Tester5	52	0.294194614	0.570881843	0.478435031	8	4	4	12	20	76.65%	1.73%	3	14
413	Tester5	53	0.285878071	0.565212335	0.485160223	9	4	4	14	22	74.64%	1.00%	4	14
414	Tester5	54	0.282476308	0.570341051	0.471627931	9	4	4	14	22	77.00%	0.00%	2	13
415	Tester5	55	0.284204796	0.560767523	0.48080234	10	5	4	12	23	75.63%	1.43%	1	14
416	Tester5	56	0.29291162	0.564947472	0.489070847	9	5	6	12	21	74.85%	1.01%	4	15
417	Tester5	57	0.286327232	0.560308738	0.482551831	9	5	5	13	21	73.85%	1.83%	3	15
418	Tester5	58	0.285681298	0.573523343	0.481094876	10	4	7	12	21	74.12%	2.35%	2	14
419	Tester5	59	0.279335936	0.566886017	0.469386851	11	4	7	12	23	74.12%	1.52%	2	15
420	Tester5	60	0.282337564	0.560242225	0.485918289	10	5	5	13	22	74.49%	1.61%	1	15
421	Tester5	61	0.290302436	0.573364598	0.490895791	12	6	7	12	19	73.77%	1.56%	3	15
422	Tester5	62	0.281958162	0.566569595	0.482808729	10	5	6	13	22	72.84%	0.96%	1	18
423	Tester5	63	0.287804844	0.566090882	0.483967385	11	4	6	12	22	72.35%	0.69%	1	16
424	Tester5	64	0.284791714	0.574882983	0.476845908	12	5	8	13	22	73.07%	1.50%	3	15
425	Tester5	65	0.291729597	0.565166996	0.480998911	11	5	5	13	21	71.18%	2.25%	1	16
426	Tester5	66	0.290710908	0.559201762	0.487265886	11	6	7	14	22	71.98%	2.52%	1	16
427	Tester5	67	0.292271936	0.560675457	0.486720022	12	5	6	11	21	72.00%	1.58%	4	17
428	Tester5	68	0.288738352	0.569797883	0.484707298	13	6	7	13	23	70.70%	0.00%	3	14
429	Tester5	69	0.28476765	0.565609718	0.482438113	12	7	6	12	24	71.94%	0.00%	2	17
430	Tester5	70	0.285900688	0.563101897	0.487360862	14	5	6	14	23	69.88%	0.08%	4	16
431	Tester5	71	0.29874284	0.570231651	0.483224413	14	5	8	13	25	71.78%	0.00%	5	16
432	Tester5	72	0.297040662	0.557155865	0.483040503	13	7	5	13	22	71.49%	1.49%	2	17
433	Tester5	73	0.286018203	0.568993131	0.480531474	14	6	6	14	24	68.56%	2.04%	3	16

434	Tester5	74	0.287949519	0.563072881	0.485978975	12	4	9	14	24	68.35%	1.74%	3	17
435	Tester5	75	0.286920848	0.559115536	0.490085736	15	7	8	14	24	69.39%	1.37%	3	17
436	Tester5	76	0.285622862	0.569507779	0.485936393	13	7	8	13	23	70.09%	3.72%	0	17
437	Tester5	77	0.290791767	0.567299709	0.480674411	15	7	9	14	24	68.22%	1.16%	3	17
438	Tester5	78	0.290027951	0.560272897	0.485560518	14	6	8	14	24	68.20%	2.11%	1	18
439	Tester5	79	0.289387067	0.564712627	0.477632062	15	7	9	13	24	68.51%	1.62%	3	18
440	Tester5	80	0.291179974	0.557494815	0.483087678	14	5	7	14	25	68.04%	1.04%	3	19
441	Tester5	81	0.287987055	0.56865852	0.475199561	16	5	8	13	24	67.82%	1.40%	2	17
442	Tester5	82	0.286593449	0.561994867	0.480519569	15	7	8	13	23	65.85%	1.76%	2	21
443	Tester5	83	0.291610046	0.570544392	0.481655986	16	7	11	14	25	67.17%	2.74%	2	19
444	Tester5	84	0.300991356	0.563278209	0.483709621	15	7	7	13	26	65.04%	1.88%	3	20
445	Tester5	85	0.288998985	0.562163178	0.474757434	18	9	7	14	25	63.66%	0.75%	2	19
446	Tester5	86	0.293380948	0.564815872	0.476847992	16	8	8	14	24	66.11%	1.89%	5	19
447	Tester5	87	0.297839094	0.568724816	0.479464255	17	7	7	14	26	64.59%	0.65%	2	20
448	Tester5	88	0.287601216	0.555901846	0.479959649	16	7	9	13	26	65.70%	4.27%	4	20
449	Tester5	89	0.285867924	0.559772864	0.481473774	18	7	8	15	25	65.69%	1.37%	4	20
450	Tester5	90	0.292948981	0.547729626	0.467409719	17	7	9	14	26	65.60%	0.40%	3	18
451	Tester6	1	0.290363701	0.520249289	0.448179696	0	2	0	15	21	88.86%	0.00%	3	8
452	Tester6	2	0.292608574	0.521139804	0.453186051	0	2	0	15	25	88.91%	0.53%	3	8
453	Tester6	3	0.288109607	0.515627305	0.456571741	0	0	0	13	24	89.43%	1.00%	2	7
454	Tester6	4	0.280432955	0.515486758	0.448898796	0	0	0	11	24	88.46%	1.58%	2	8
455	Tester6	5	0.290858286	0.521690211	0.456623805	0	0	0	12	24	89.00%	0.00%	2	8
456	Tester6	6	0.289212648	0.525929324	0.453423035	0	2	0	14	24	87.96%	0.00%	2	9
457	Tester6	7	0.296468134	0.5187393	0.459172169	0	0	0	12	24	86.20%	0.55%	3	9
458	Tester6	8	0.294725891	0.529926406	0.465825382	0	0	1	11	23	87.66%	0.37%	2	8
459	Tester6	9	0.285252382	0.528917147	0.450997753	1	1	0	13	23	85.95%	0.00%	2	7
460	Tester6	10	0.288744417	0.52571744	0.451590611	1	1	1	14	22	85.65%	0.84%	2	8
461	Tester6	11	0.285882179	0.537421986	0.456859096	2	1	1	11	24	86.68%	0.05%	2	8
462	Tester6	12	0.281306505	0.527256812	0.46163037	3	2	1	11	23	87.10%	0.00%	2	7
463	Tester6	13	0.293304331	0.531497456	0.457731324	1	0	0	10	21	86.52%	0.46%	3	10
464	Tester6	14	0.295938273	0.536973973	0.459438659	3	4	2	11	23	87.35%	0.00%	4	9
465	Tester6	15	0.279279897	0.543861573	0.453290606	3	2	3	11	24	86.46%	1.88%	1	9
466	Tester6	16	0.293813364	0.539555964	0.469702045	4	1	2	13	22	84.78%	0.00%	0	9
467	Tester6	17	0.293772258	0.547035284	0.472273683	3	3	0	13	22	84.40%	2.11%	3	10
468	Tester6	18	0.287773782	0.54289132	0.452000511	3	3	1	12	23	86.42%	0.00%	2	10
469	Tester6	19	0.294523862	0.539704898	0.464858543	4	0	2	15	20	83.42%	0.59%	2	9
470	Tester6	20	0.285505671	0.541259406	0.47120483	0	1	1	12	22	85.96%	0.00%	3	9
471	Tester6	21	0.290821241	0.547806903	0.471088486	2	3	1	12	22	84.27%	0.00%	4	11
472	Tester6	22	0.288145824	0.552029168	0.459689585	3	2	2	11	21	84.26%	0.99%	3	9
473	Tester6	23	0.286860194	0.552234297	0.466707527	2	1	3	13	21	83.40%	0.28%	1	10
474	Tester6	24	0.289443663	0.549079067	0.473477017	4	1	3	14	20	82.82%	1.47%	2	11
475	Tester6	25	0.297136162	0.552946685	0.462382636	4	1	0	12	22	86.06%	0.29%	4	10
476	Tester6	26	0.294671542	0.551611595	0.466555905	5	2	2	12	22	83.43%	1.77%	2	8
477	Tester6	27	0.291609041	0.550247705	0.481655549	4	0	3	12	20	84.34%	0.68%	0	11
478	Tester6	28	0.289375385	0.556184618	0.470030949	5	3	3	12	19	81.58%	0.89%	2	12
479	Tester6	29	0.296175633	0.553343735	0.478852407	5	4	3	12	20	82.65%	0.03%	3	11
480	Tester6	30	0.286683131	0.560319103	0.473490988	5	2	2	12	21	82.01%	1.87%	3	11
481	Tester6	31	0.284464814	0.552128788	0.472887086	4	3	5	11	22	80.93%	0.47%	3	11
482	Tester6	32	0.288233017	0.555063828	0.465467106	5	2	3	11	22	82.45%	3.71%	2	13
483	Tester6	33	0.288744332	0.560190487	0.468472526	5	2	4	12	18	81.63%	0.85%	2	10
484	Tester6	34	0.291592887	0.560996802	0.473860098	5	3	4	10	21	79.66%	1.69%	3	11
485	Tester6	35	0.293073193	0.560016811	0.474629589	6	3	4	11	20	80.30%	0.00%	4	12
486	Tester6	36	0.281552157	0.555642049	0.469746282	4	2	4	12	21	79.34%	1.33%	3	14
487	Tester6	37	0.292407145	0.555499507	0.470964323	8	3	4	13	21	79.11%	0.00%	3	11
488	Tester6	38	0.291198515	0.550031021	0.478307099	7	4	4	11	22	79.13%	0.00%	2	12
489	Tester6	39	0.295200971	0.561472007	0.469665262	8	4	5	11	22	79.47%	0.00%	2	11
490	Tester6	40	0.294755562	0.567723862	0.484815091	6	2	4	11	21	80.24%	0.94%	4	11
491	Tester6	41	0.289951912	0.561104813	0.484723656	9	2	3	14	22	79.73%	0.44%	2	14
492	Tester6	42	0.282801024	0.56720682	0.477613835	7	1	2	11	22	78.77%	0.00%	2	13
493	Tester6	43	0.287720606	0.564213059	0.480186588	8	5	4	10	21	78.36%	0.00%	6	12
494	Tester6	44	0.287223354	0.56355641	0.475272179	7	3	4	11	21	77.57%	0.99%	3	14
495	Tester6	45	0.293460909	0.574792712	0.485014367	7	2	3	13	20	78.54%	0.14%	2	10

496	Tester6	46	0.297388764	0.562926653	0.48201375	7	5	4	11	22	75.86%	0.62%	3	13
497	Tester6	47	0.281638385	0.555489243	0.477629718	6	2	4	13	21	77.29%	1.61%	3	11
498	Tester6	48	0.290491012	0.565042662	0.483712337	9	4	3	13	22	75.67%	0.00%	2	14
499	Tester6	49	0.293235897	0.563238375	0.484226745	8	4	5	10	23	76.83%	0.07%	3	14
500	Tester6	50	0.28571328	0.56676413	0.48009136	9	2	3	13	20	76.42%	1.82%	4	15
501	Tester6	51	0.288545155	0.564836371	0.482984375	6	4	5	11	22	75.56%	1.80%	2	14
502	Tester6	52	0.290958276	0.567259751	0.472460917	10	5	3	13	21	73.48%	1.84%	2	13
503	Tester6	53	0.29187733	0.574200925	0.485972398	10	4	4	10	20	73.73%	0.25%	4	14
504	Tester6	54	0.292554817	0.574978374	0.478657544	10	5	3	11	20	73.75%	0.31%	3	14
505	Tester6	55	0.29166277	0.56318937	0.478177069	11	6	6	12	21	76.01%	1.35%	2	14
506	Tester6	56	0.290335943	0.56544823	0.484811654	11	4	5	11	22	74.13%	1.20%	4	15
507	Tester6	57	0.286405604	0.568095262	0.487619061	9	3	5	12	23	73.48%	2.05%	3	15
508	Tester6	58	0.293552511	0.567342415	0.482908008	11	5	6	12	23	73.81%	5.34%	1	15
509	Tester6	59	0.289364215	0.559927977	0.485455061	10	2	4	10	21	73.55%	1.37%	2	14
510	Tester6	60	0.293388765	0.561708986	0.477046393	11	4	6	12	21	73.86%	0.51%	2	15
511	Tester6	61	0.287508401	0.574621114	0.482449713	10	4	6	13	22	74.09%	3.77%	3	16
512	Tester6	62	0.285171039	0.56928496	0.478433068	10	5	8	11	21	73.89%	0.97%	3	15
513	Tester6	63	0.289160355	0.568824495	0.491689529	13	5	5	12	22	72.31%	1.34%	4	16
514	Tester6	64	0.285111991	0.563807976	0.480795617	11	7	5	13	23	72.44%	2.53%	3	15
515	Tester6	65	0.292674052	0.560120236	0.478260244	11	4	6	13	24	74.02%	0.29%	3	15
516	Tester6	66	0.289593498	0.562541188	0.481746475	13	5	5	12	23	73.11%	2.38%	3	14
517	Tester6	67	0.290979454	0.567967769	0.491207752	11	5	5	13	22	71.10%	0.00%	4	17
518	Tester6	68	0.297808328	0.567119264	0.486840923	12	6	7	13	23	72.33%	0.86%	2	17
519	Tester6	69	0.28584714	0.559238377	0.481515866	12	5	8	13	23	71.10%	1.81%	2	16
520	Tester6	70	0.284947202	0.57096726	0.48045619	14	6	7	12	23	69.79%	1.43%	2	15
521	Tester6	71	0.285734831	0.569771964	0.480753277	13	6	7	13	23	71.57%	0.81%	4	17
522	Tester6	72	0.287227962	0.574613248	0.482602753	13	9	7	14	24	70.38%	2.29%	2	17
523	Tester6	73	0.288728701	0.568043172	0.484651375	14	5	9	13	22	70.22%	2.06%	0	17
524	Tester6	74	0.289596396	0.554182206	0.482496302	17	5	5	14	24	68.62%	2.55%	2	17
525	Tester6	75	0.285276632	0.565157152	0.480637378	13	6	7	13	24	69.17%	2.00%	2	19
526	Tester6	76	0.289030384	0.566034569	0.481808353	13	5	8	14	24	68.83%	3.89%	4	17
527	Tester6	77	0.288882624	0.554703359	0.474807292	15	7	7	14	24	68.64%	3.12%	3	19
528	Tester6	78	0.294493041	0.564319873	0.484092299	14	6	6	13	25	68.11%	2.51%	2	17
529	Tester6	79	0.29253254	0.558341138	0.478723042	16	7	7	12	25	68.07%	1.19%	3	17
530	Tester6	80	0.282815076	0.563327455	0.488083128	17	6	10	14	25	67.35%	2.23%	2	17
531	Tester6	81	0.291980007	0.550695392	0.478451483	15	7	9	13	24	67.09%	0.68%	2	17
532	Tester6	82	0.289456865	0.561097839	0.48681343	17	6	9	13	24	67.70%	1.14%	3	19
533	Tester6	83	0.291117793	0.558659809	0.477696977	15	7	7	13	24	66.17%	2.55%	2	17
534	Tester6	84	0.288983169	0.565489826	0.475599709	17	7	7	14	27	66.98%	1.62%	2	18
535	Tester6	85	0.293693714	0.572241675	0.485680083	16	6	10	15	25	67.14%	1.72%	1	18
536	Tester6	86	0.289445447	0.561208929	0.476493774	17	6	8	15	24	64.90%	1.94%	4	19
537	Tester6	87	0.291395188	0.554767427	0.484755528	18	7	10	16	25	66.23%	3.61%	4	19
538	Tester6	88	0.286753976	0.555235505	0.475407822	18	9	8	14	27	62.76%	2.64%	4	20
539	Tester6	89	0.291738352	0.564614821	0.474438571	17	7	7	14	26	65.20%	2.80%	4	20
540	Tester6	90	0.285137296	0.555262083	0.476899065	18	8	9	14	27	66.22%	1.67%	3	20
541	Tester7	1	0.289192863	0.526319066	0.451834758	0	0	0	12	24	89.81%	0.97%	2	7
542	Tester7	2	0.296449399	0.527414538	0.458562645	1	1	0	14	23	89.28%	0.66%	3	8
543	Tester7	3	0.287063274	0.508452028	0.44810446	2	1	1	12	22	90.95%	0.29%	4	8
544	Tester7	4	0.29629481	0.519780193	0.446940167	2	0	0	14	23	89.27%	0.34%	3	10
545	Tester7	5	0.290998494	0.522012982	0.448072315	0	1	0	14	23	88.87%	1.91%	3	9
546	Tester7	6	0.294623935	0.519971041	0.454663645	0	1	0	12	23	89.15%	1.06%	2	8
547	Tester7	7	0.295421401	0.529762507	0.454968189	0	0	3	13	22	88.58%	0.58%	2	8
548	Tester7	8	0.289822668	0.522535026	0.454758389	0	2	0	13	23	87.95%	0.00%	3	10
549	Tester7	9	0.288072206	0.53324993	0.457761515	2	0	1	13	23	87.27%	0.91%	3	9
550	Tester7	10	0.298394122	0.536529389	0.458938457	1	0	1	13	20	86.99%	0.62%	2	8
551	Tester7	11	0.296232885	0.540440621	0.452006533	3	0	0	12	23	86.06%	0.00%	3	9
552	Tester7	12	0.292287252	0.533494257	0.463370261	1	1	1	13	23	88.98%	0.17%	3	11
553	Tester7	13	0.287168679	0.540865625	0.45534334	0	0	1	11	21	86.92%	1.03%	2	8
554	Tester7	14	0.292514518	0.53832215	0.463264519	0	0	0	11	21	85.77%	1.83%	3	9
555	Tester7	15	0.286865548	0.534784361	0.4629147	2	2	0	13	23	87.61%	0.00%	2	9
556	Tester7	16	0.287539373	0.534466433	0.466208199	1	1	1	12	20	85.93%	0.00%	1	7
557	Tester7	17	0.290176552	0.536582981	0.457525183	1	1	1	14	21	85.24%	0.00%	1	10

558	Tester7	18	0.28962981	0.539743401	0.475246673	3	2	3	12	23	85.64%	0.00%	4	8
559	Tester7	19	0.290011514	0.55092162	0.46372014	3	2	1	14	21	85.07%	0.00%	1	9
560	Tester7	20	0.298229599	0.541470395	0.464877248	3	1	1	13	22	83.58%	0.00%	1	9
561	Tester7	21	0.295184894	0.54901666	0.468036401	4	1	0	12	21	83.88%	1.29%	2	9
562	Tester7	22	0.290037888	0.553680116	0.457897504	1	2	2	13	24	83.54%	0.75%	2	10
563	Tester7	23	0.292588597	0.545000238	0.477195393	5	2	3	13	20	83.61%	0.95%	4	11
564	Tester7	24	0.287375419	0.549160218	0.470757797	3	2	1	12	22	85.01%	0.20%	4	10
565	Tester7	25	0.289095382	0.545841886	0.475068671	1	3	0	13	19	84.46%	0.00%	2	10
566	Tester7	26	0.283069109	0.551245342	0.47476743	5	1	3	13	21	82.26%	0.84%	3	10
567	Tester7	27	0.29292175	0.549413362	0.473381674	4	3	0	13	21	82.78%	0.09%	2	10
568	Tester7	28	0.278407294	0.549927799	0.472632959	5	3	2	11	23	82.70%	0.02%	3	10
569	Tester7	29	0.291188077	0.551195016	0.486401803	3	2	2	13	22	81.08%	0.65%	4	11
570	Tester7	30	0.283932116	0.546947668	0.467481352	4	2	3	12	20	81.49%	1.78%	1	9
571	Tester7	31	0.28536549	0.553222007	0.478578762	5	2	3	11	22	80.65%	0.00%	2	9
572	Tester7	32	0.287784709	0.550511837	0.476577814	5	3	4	12	20	81.85%	0.00%	1	11
573	Tester7	33	0.288601531	0.554306029	0.465930486	5	2	2	12	21	81.16%	0.86%	3	10
574	Tester7	34	0.283505807	0.559362062	0.484310205	5	1	3	12	21	80.14%	0.01%	1	12
575	Tester7	35	0.28610742	0.555886515	0.474154516	5	1	2	11	20	79.59%	0.00%	2	11
576	Tester7	36	0.277983784	0.556683723	0.468387846	7	2	4	11	21	79.91%	0.77%	2	10
577	Tester7	37	0.28416444	0.561587504	0.470040832	6	2	2	12	20	79.97%	0.63%	4	11
578	Tester7	38	0.287751175	0.563422996	0.477696914	7	2	3	11	23	78.73%	0.02%	4	10
579	Tester7	39	0.291047477	0.555066529	0.489587643	6	3	3	12	19	80.69%	0.00%	1	13
580	Tester7	40	0.285732742	0.553706508	0.469856698	7	3	3	12	22	78.40%	2.37%	4	12
581	Tester7	41	0.281376225	0.560579694	0.482912607	8	2	4	13	20	79.68%	0.00%	3	12
582	Tester7	42	0.298259448	0.55768758	0.479878067	6	3	5	13	21	77.91%	1.65%	2	13
583	Tester7	43	0.295431515	0.563937674	0.48832842	6	0	3	11	21	77.94%	0.00%	2	13
584	Tester7	44	0.288404653	0.563163857	0.486263892	8	1	4	12	22	77.02%	0.60%	2	12
585	Tester7	45	0.286090712	0.564412567	0.476012602	7	4	4	14	21	78.13%	0.67%	2	13
586	Tester7	46	0.281798445	0.564466347	0.47898849	8	5	4	11	20	76.06%	1.03%	2	13
587	Tester7	47	0.300751934	0.567386788	0.484888773	6	2	5	12	20	79.47%	0.68%	4	13
588	Tester7	48	0.288713725	0.56744574	0.49121576	8	4	5	12	20	77.83%	0.00%	4	14
589	Tester7	49	0.282554194	0.564064313	0.483037003	8	5	5	11	20	77.15%	0.00%	2	14
590	Tester7	50	0.28487688	0.566141563	0.481776081	9	4	6	11	21	74.67%	2.28%	4	14
591	Tester7	51	0.28542302	0.563491951	0.481410348	9	2	5	11	21	76.65%	2.22%	2	14
592	Tester7	52	0.285526799	0.552647053	0.481340671	7	3	5	13	21	76.84%	0.80%	1	14
593	Tester7	53	0.288722397	0.567337012	0.478798417	8	3	5	13	22	75.85%	0.55%	3	14
594	Tester7	54	0.285662952	0.562029315	0.484160024	9	4	7	12	21	75.11%	1.24%	4	14
595	Tester7	55	0.286495742	0.562463365	0.484344726	10	2	5	12	22	73.19%	0.89%	3	14
596	Tester7	56	0.293478368	0.573647284	0.479243262	9	5	6	13	22	74.26%	1.03%	2	14
597	Tester7	57	0.290669131	0.573836689	0.481626839	10	2	6	14	20	74.96%	0.30%	1	13
598	Tester7	58	0.288300533	0.562055759	0.488934422	10	5	6	11	22	75.97%	2.05%	4	13
599	Tester7	59	0.290782661	0.565960708	0.489938716	10	5	6	12	22	73.64%	0.00%	2	15
600	Tester7	60	0.289212274	0.570550131	0.485365281	12	4	6	14	22	73.70%	0.79%	2	14
601	Tester7	61	0.292451502	0.562867448	0.475852132	12	4	6	12	22	74.39%	1.98%	1	16
602	Tester7	62	0.296263271	0.56478644	0.481135008	11	5	4	12	22	73.88%	0.00%	0	15
603	Tester7	63	0.280963806	0.563346698	0.479144741	11	5	7	12	21	72.60%	2.06%	1	17
604	Tester7	64	0.288043436	0.569613146	0.477754704	12	4	6	10	21	72.11%	0.54%	2	16
605	Tester7	65	0.291306607	0.565133243	0.482015236	13	4	5	13	22	73.02%	1.91%	3	15
606	Tester7	66	0.282100398	0.568460437	0.488499826	12	5	5	12	23	73.74%	2.29%	3	17
607	Tester7	67	0.287319603	0.562374714	0.489389182	13	5	8	13	21	71.56%	0.76%	2	17
608	Tester7	68	0.285561185	0.560098026	0.485822589	13	4	6	14	22	72.15%	0.86%	3	15
609	Tester7	69	0.291564327	0.569985247	0.481209925	12	5	6	11	22	70.53%	1.91%	3	16
610	Tester7	70	0.291491562	0.562313271	0.481805422	12	4	6	13	23	71.06%	1.80%	2	18
611	Tester7	71	0.302977269	0.566784461	0.486115935	11	6	6	13	23	69.22%	2.22%	2	16
612	Tester7	72	0.289721349	0.56114342	0.476866853	12	7	7	13	24	69.85%	0.91%	2	18
613	Tester7	73	0.287267304	0.558364989	0.487588377	12	5	7	13	21	70.05%	0.87%	2	17
614	Tester7	74	0.293080941	0.556469904	0.487809333	13	7	7	14	23	69.15%	1.41%	3	18
615	Tester7	75	0.293262519	0.560861801	0.476137575	14	5	8	14	23	68.45%	2.10%	2	17
616	Tester7	76	0.288298967	0.564275041	0.478850813	15	6	8	12	24	68.36%	1.12%	3	18
617	Tester7	77	0.287463342	0.558403257	0.481024865	14	6	9	15	22	67.80%	3.23%	2	17
618	Tester7	78	0.288986695	0.558812571	0.484913563	13	8	6	12	24	69.20%	1.81%	3	19
619	Tester7	79	0.28902962	0.563062372	0.479707091	15	7	8	14	24	69.47%	3.97%	2	18

620	Tester7	80	0.282626558	0.554642726	0.484330218	15	5	8	12	25	67.39%	2.30%	3	17
621	Tester7	81	0.28327114	0.556878193	0.484539137	16	7	8	12	27	69.28%	2.84%	2	17
622	Tester7	82	0.283374247	0.561033709	0.489812853	15	6	8	14	25	65.84%	3.06%	1	20
623	Tester7	83	0.292502616	0.565342523	0.476045715	17	6	8	15	26	65.57%	1.31%	4	20
624	Tester7	84	0.291964619	0.560787948	0.484224518	18	9	7	14	25	68.41%	2.80%	3	20
625	Tester7	85	0.292881859	0.563628887	0.473984919	16	7	6	14	25	65.54%	1.32%	4	19
626	Tester7	86	0.290523961	0.5643139	0.474894767	16	7	8	14	26	68.03%	2.23%	2	19
627	Tester7	87	0.294282435	0.560747156	0.471736529	19	8	10	13	27	64.09%	0.27%	3	19
628	Tester7	88	0.290939489	0.553646863	0.482163036	16	8	10	14	25	65.26%	2.52%	4	19
629	Tester7	89	0.289627919	0.5532484	0.476452965	17	9	8	15	26	65.20%	2.01%	3	20
630	Tester7	90	0.294660558	0.568267483	0.479503508	17	8	8	15	25	64.14%	2.64%	1	22
631	Tester8	1	0.289247719	0.512155044	0.450595917	1	2	1	14	24	88.98%	0.00%	1	7
632	Tester8	2	0.292132349	0.522941192	0.454984935	0	0	2	13	24	90.84%	2.85%	3	8
633	Tester8	3	0.291339543	0.517155046	0.454863875	0	0	0	15	24	88.14%	0.00%	1	8
634	Tester8	4	0.292937386	0.522605213	0.452214886	0	1	1	13	23	91.17%	0.92%	3	8
635	Tester8	5	0.291113118	0.522368567	0.448416505	0	2	0	12	22	88.49%	0.00%	4	9
636	Tester8	6	0.285472979	0.530529282	0.455440465	1	1	1	14	22	89.52%	0.00%	1	8
637	Tester8	7	0.28851286	0.532900972	0.45154455	0	0	2	15	23	87.50%	0.56%	3	8
638	Tester8	8	0.297798969	0.526372279	0.450731241	2	3	0	12	22	89.25%	0.00%	3	7
639	Tester8	9	0.295023981	0.516632177	0.447270593	1	1	1	12	22	86.92%	0.00%	2	10
640	Tester8	10	0.279736856	0.530150667	0.457613048	0	1	0	13	23	87.10%	0.00%	3	11
641	Tester8	11	0.287492083	0.53489572	0.45728981	1	1	1	13	23	86.36%	0.04%	2	8
642	Tester8	12	0.288701214	0.535542513	0.449497477	0	2	0	13	23	86.83%	0.00%	1	10
643	Tester8	13	0.288767387	0.529716709	0.459676258	1	2	1	13	22	85.16%	1.33%	3	10
644	Tester8	14	0.287232526	0.537375337	0.461590281	0	1	1	10	20	85.36%	0.00%	3	9
645	Tester8	15	0.289886094	0.541904152	0.462223135	3	1	2	13	21	85.62%	0.14%	3	10
646	Tester8	16	0.293555301	0.535587251	0.467316929	2	2	2	12	21	86.84%	1.61%	2	8
647	Tester8	17	0.28957107	0.53314997	0.464361971	4	2	0	12	23	87.87%	0.25%	2	9
648	Tester8	18	0.294884258	0.534638895	0.460624048	2	1	1	14	21	85.50%	0.00%	1	10
649	Tester8	19	0.287750988	0.546702359	0.469633585	1	1	1	13	21	85.88%	0.00%	4	9
650	Tester8	20	0.289304055	0.54222741	0.471192893	1	2	1	14	22	84.87%	1.49%	3	10
651	Tester8	21	0.294411905	0.546474921	0.465329032	3	1	3	12	21	83.89%	0.04%	1	8
652	Tester8	22	0.293071909	0.546340296	0.464191126	3	2	3	12	23	84.29%	0.76%	3	9
653	Tester8	23	0.292091756	0.542359533	0.47365892	2	3	1	11	20	83.17%	0.00%	2	9
654	Tester8	24	0.288837973	0.544239516	0.473348283	3	1	1	13	20	84.57%	1.40%	2	11
655	Tester8	25	0.284836367	0.547001992	0.475073234	3	1	1	11	21	81.33%	0.52%	1	9
656	Tester8	26	0.286750675	0.547191846	0.475718853	4	4	2	12	21	83.80%	2.74%	2	11
657	Tester8	27	0.294236853	0.538114968	0.468545047	5	2	2	13	19	82.74%	1.26%	3	11
658	Tester8	28	0.287599505	0.548736698	0.47102527	3	2	2	11	21	84.64%	0.27%	2	11
659	Tester8	29	0.282864261	0.565176523	0.474644973	5	2	3	13	19	82.61%	1.03%	3	10
660	Tester8	30	0.293840235	0.558592587	0.479388411	5	2	3	12	22	83.16%	1.22%	2	10
661	Tester8	31	0.29737094	0.554312924	0.471506419	4	3	2	12	21	83.03%	0.67%	4	12
662	Tester8	32	0.284688598	0.553898203	0.475788742	5	3	4	13	22	81.86%	2.70%	2	11
663	Tester8	33	0.283014145	0.557243522	0.462008956	6	2	2	11	20	81.40%	0.72%	2	13
664	Tester8	34	0.295384265	0.552900763	0.481065952	6	2	3	12	20	80.56%	0.68%	3	12
665	Tester8	35	0.292332988	0.548181883	0.476874838	7	2	4	10	22	81.32%	0.00%	3	11
666	Tester8	36	0.294384869	0.554210809	0.481072347	7	3	4	12	21	80.66%	1.45%	4	10
667	Tester8	37	0.286867581	0.56043328	0.483183977	5	3	3	10	22	82.20%	0.68%	4	13
668	Tester8	38	0.284508979	0.560584077	0.483330678	4	4	1	11	21	78.70%	0.86%	2	12
669	Tester8	39	0.294807708	0.563882179	0.476314458	7	2	5	13	21	80.51%	0.55%	2	13
670	Tester8	40	0.296592797	0.556658828	0.476895359	7	3	4	12	22	79.84%	0.33%	1	12
671	Tester8	41	0.294260104	0.560874811	0.47649619	6	3	4	13	22	78.77%	1.80%	2	11
672	Tester8	42	0.286966565	0.564291813	0.481533645	7	3	4	13	20	77.20%	0.67%	4	14
673	Tester8	43	0.289716607	0.555484041	0.482452327	5	3	3	11	22	77.45%	2.06%	2	11
674	Tester8	44	0.28472543	0.554846153	0.485196914	8	3	5	10	21	78.18%	0.00%	2	13
675	Tester8	45	0.285446708	0.568452504	0.478864784	6	2	3	12	22	76.48%	0.53%	3	14
676	Tester8	46	0.28377626	0.56940317	0.474517642	9	3	3	11	21	77.28%	0.27%	3	13
677	Tester8	47	0.286522111	0.555078452	0.482778222	8	4	5	12	19	74.67%	1.98%	3	12
678	Tester8	48	0.284402503	0.571030314	0.478930251	7	3	4	11	21	78.83%	0.00%	1	13
679	Tester8	49	0.287631398	0.559692538	0.478089998	8	3	4	12	22	77.38%	0.00%	3	14
680	Tester8	50	0.28737737	0.568266223	0.486079673	8	4	5	12	21	76.85%	0.00%	1	13
681	Tester8	51	0.279174691	0.558234185	0.48492425	8	4	6	12	22	76.56%	0.00%	3	14

682	Tester8	52	0.287298166	0.576677008	0.486407145	9	3	4	11	20	75.16%	0.00%	2	13
683	Tester8	53	0.287716394	0.569804405	0.482607294	9	4	4	12	21	74.92%	2.61%	2	15
684	Tester8	54	0.284152535	0.57081049	0.4848401	8	5	6	13	22	76.84%	1.78%	1	14
685	Tester8	55	0.295317958	0.558667324	0.482633454	12	5	6	12	22	75.40%	0.15%	2	15
686	Tester8	56	0.287065334	0.560636914	0.483130717	10	4	5	14	23	74.20%	0.68%	2	13
687	Tester8	57	0.285379121	0.566258818	0.480811206	9	5	4	14	22	73.94%	1.18%	2	14
688	Tester8	58	0.28427041	0.569842706	0.48949264	10	5	5	13	24	75.39%	2.05%	2	15
689	Tester8	59	0.287354775	0.56472826	0.487174689	10	6	3	14	23	74.01%	1.60%	1	16
690	Tester8	60	0.290987638	0.56273785	0.483480635	10	4	7	13	22	73.47%	1.70%	4	15
691	Tester8	61	0.280287408	0.56899035	0.485592006	10	3	8	12	22	73.79%	1.49%	2	15
692	Tester8	62	0.29510085	0.562382943	0.492472657	12	6	7	13	23	73.83%	0.30%	4	17
693	Tester8	63	0.285405971	0.572570424	0.482416801	10	6	7	12	24	73.71%	0.00%	3	15
694	Tester8	64	0.285457364	0.55911878	0.479739583	12	5	6	12	21	71.20%	1.55%	3	14
695	Tester8	65	0.281147783	0.565892934	0.481256363	12	4	6	13	22	70.91%	0.26%	2	15
696	Tester8	66	0.285901752	0.568762682	0.484552364	11	4	5	12	22	70.17%	1.57%	2	16
697	Tester8	67	0.289359696	0.569101591	0.471875822	13	4	7	12	21	71.70%	0.00%	2	18
698	Tester8	68	0.289890722	0.563855045	0.485382244	11	4	7	12	22	71.04%	1.89%	2	16
699	Tester8	69	0.281516434	0.562713988	0.484981738	12	6	7	12	21	71.05%	2.50%	2	18
700	Tester8	70	0.289829574	0.565232582	0.481548224	12	6	8	14	24	72.45%	1.44%	0	17
701	Tester8	71	0.281347258	0.56387381	0.488870261	14	5	7	11	22	69.97%	1.12%	2	17
702	Tester8	72	0.294347789	0.563482543	0.477191897	12	6	6	14	22	70.52%	2.31%	2	16
703	Tester8	73	0.278739636	0.565964215	0.491301757	13	7	7	13	21	69.19%	1.23%	3	17
704	Tester8	74	0.280991612	0.566650282	0.476194742	14	7	8	11	23	67.29%	2.96%	1	17
705	Tester8	75	0.283602454	0.562484456	0.479272976	14	6	8	12	23	67.23%	1.02%	1	18
706	Tester8	76	0.288383987	0.558214872	0.476841668	13	5	7	13	24	68.16%	1.52%	2	17
707	Tester8	77	0.28847413	0.564583632	0.484124156	15	9	9	12	23	68.31%	2.60%	3	18
708	Tester8	78	0.278533356	0.558432097	0.480503089	14	7	8	15	24	67.74%	1.53%	3	17
709	Tester8	79	0.292528869	0.564217303	0.475247168	14	7	7	12	24	68.33%	2.93%	4	18
710	Tester8	80	0.282546099	0.555464481	0.483359858	16	5	8	14	25	68.16%	2.12%	2	19
711	Tester8	81	0.287559736	0.563146948	0.476786871	16	8	7	14	25	65.72%	2.26%	5	16
712	Tester8	82	0.289554319	0.557128511	0.48460417	15	8	10	14	25	66.32%	2.73%	4	19
713	Tester8	83	0.293862923	0.562208935	0.484987212	17	7	10	15	25	67.66%	2.97%	4	19
714	Tester8	84	0.292445261	0.56326618	0.469231586	14	7	9	14	26	66.85%	1.26%	1	18
715	Tester8	85	0.291553424	0.557496009	0.474738613	17	7	8	13	27	67.09%	1.78%	3	17
716	Tester8	86	0.299505156	0.569564451	0.474812261	19	7	9	14	27	66.67%	1.40%	3	18
717	Tester8	87	0.29989573	0.549781481	0.478925266	17	7	8	16	26	64.40%	1.87%	2	20
718	Tester8	88	0.286610799	0.562259358	0.47108683	17	8	8	14	25	65.13%	3.42%	2	20
719	Tester8	89	0.293434842	0.555595273	0.475401444	17	7	8	15	26	65.51%	1.01%	3	19
720	Tester8	90	0.279946975	0.560465813	0.480694972	17	9	8	15	28	62.73%	1.92%	4	22
