# Supplementary material for: A numerical evaluation of real-time workloads for ramp controller through optimization of multi-type feature combinations derived from eye tracker, respiratory, and fatigue patterns
Source: PLoS One. 2024 Nov 8;19(11):e0313565. doi: 10.1371/journal.pone.0313565 (PMC11548742; doi:10.1371/journal.pone.0313565)
Supplement: Attachment — Submitted filename: Response to Reviewers.docx [file pone.0313565.s002.docx]

**Response to reviewers on the paper entitled “A numerical evaluation of real-time workloads for ramp controller through optimization of multi-type feature combinations derived from eye tracker, respiratory, and fatigue patterns”**

*Manuscript ID:* *PONE-D-24-23960*

*Journal: PLOS ONE*

*Revision: R1*

We would like to express our gratitude and appreciation to the reviewers for providing valuable comments to ensure our manuscript’s continuing improvement. All the comments provided by the different reviewers have been acted upon to improve the manuscript. We hope that the editors and reviewers find this revision an improvement.

In accordance with the comments from the editors and reviewers, amendments are made as given below:

Editor:

| Editor’s comment | Reply from the authors |
| --- | --- |
| Thank you for submitting your manuscript to PLOS ONE. After careful consideration, we feel that it has merit but does not fully meet PLOS ONE’s publication criteria as it currently stands. Therefore, we invite you to submit a revised version of the manuscript that addresses the points raised during the review process. | Thank you for considering our manuscript for publication in PLOS ONE. We acknowledged the comments from the reviewers and addressed them item-by-item. We hope the editors and the reviewers would find the current version of the manuscript improved. The revised content was highlighted in the manuscript in yellow. The line numbers noted below refer to the 'Revised Manuscript with Track Changes' file labeled. |
| 1.When submitting your revision, we need you to address these additional requirements. Please ensure that your manuscript meets PLOS ONE's style requirements, including those for file naming. | Thanks for your kind reminder. We have checked and confirmed our submissions align with the requirements of PLOS ONE publication. |
| 2. Thank you for stating the following financial disclosure: "This work was supported by National Natural Science Foundation of China-Civil Aviation Administration of China Civil Aviation Joint Research Fund Project (U2233208)."  Please state what role the funders took in the study. If the funders had no role, please state: "The funders had no role in study design, data collection and analysis, decision to publish, or preparation of the manuscript."  If this statement is not correct you must amend it as needed.  Please include this amended Role of Funder statement in your cover letter; we will change the online submission form on your behalf. | Thank you for your feedback. We have clarified the role of the funders as follows: The funders were involved in supervision, idea formulation, manuscript concept development, manuscript review, and revisions. This information has been added to the cover letter. |
| 3. Thank you for stating the following in the Acknowledgments Section of your manuscript: "This work was supported by National Natural Science Foundation of China-Civil Aviation Administration of China Civil Aviation Joint Research Fund Project (U2233208)."  We note that you have provided funding information that is not currently declared in your Funding Statement. However, funding information should not appear in the Acknowledgments section or other areas of your manuscript. We will only publish funding information present in the Funding Statement section of the online submission form.  Please remove any funding-related text from the manuscript and let us know how you would like to update your Funding Statement. Please include your amended statements within your cover letter; we will change the online submission form on your behalf. | Thank you for your suggestion. We have removed the Acknowledgments Section of the manuscript because this section of the original text contained only information about the fundings.  Our preferred Funding Statement is "This work was supported by National Natural Science Foundation of China-Civil Aviation Administration of China Civil Aviation Joint Research Fund Project (U2233208)." We have added this statement in the cover letter. The revised cover letter has been re-uploaded. |
| 4. PLOS requires an ORCID iD for the corresponding author in Editorial Manager on papers submitted after December 6th, 2016. Please ensure that you have an ORCID iD and that it is validated in Editorial Manager. To do this, go to ‘Update my Information’ (in the upper left-hand corner of the main menu), and click on the Fetch/Validate link next to the ORCID field. This will take you to the ORCID site and allow you to create a new iD or authenticate a pre-existing iD in Editorial Manager. | Thank you for your comment. We have activated Shao Quan's ORCID iD in this Editorial Manager account. |
| 5. Please amend your authorship list in your manuscript file to include author Dr. Ruoheng Li. | Thank you for the reminder. We have refined the author information in the manuscript. |
| 6. Please include your full ethics statement in the ‘Methods’ section of your manuscript file. In your statement, please include the full name of the IRB or ethics committee who approved or waived your study, as well as whether or not you obtained informed written or verbal consent. If consent was waived for your study, please include this information in your statement as well. | Thank you for the reminder. We have included a full ethical statement in the "Methods" section of the manuscript, including the full name of the IRB and a statement that informed written consent was obtained. |
| 7. We note that Figures 2 and 4 includes an image of a participant in the study. Please download the Consent Form for Publication in a PLOS Journal (http://journals.plos.org/plosone/s/file?id=8ce6/plos-consent-form-english.pdf). The signed consent form should not be submitted with the manuscript, but should be securely filed in the individual's case notes. Please amend the methods section and ethics statement of the manuscript to explicitly state that the patient/participant has provided consent for publication: “The individual in this manuscript has given written informed consent (as outlined in PLOS consent form) to publish these case details”. | Thank you for the reminder. The experimental participants in Figure2 and Figure4 have signed the Consent Form for Publication in a PLOS Journal. The methods section (added in lines 290-292) has been supplemented with the explicit statement that "The individual in this manuscript has given written informed consent (as outlined in PLOS consent form) to publish these case details. " |
| 8. Please include captions for your Supporting Information files at the end of your manuscript, and update any in-text citations to match accordingly. | Thank you for your comment. We have inserted an in-text citation (line 443) for Supporting Information documents and listed them at the end of the manuscript (line 1172). |

Reviewer #1:

| Reviewer’s comment | Reply from the authors |
| --- | --- |
| The authors present a study on adopting feature selection techniques with classifiers to evaluate ramp controllers' real-time workloads. The paper has provided a lot of fundamental concepts around the background, motivation, and adopted techniques. | Thanks for your efforts and time in reviewing our manuscript. Your comments have assisted us in improving the quality of our manuscript thoroughly. |
| 1. In the introduction section, there are many duplicative sentences, and they can be merged into one. For example, lines 61 to 65, 72 to 76, 89 to 94, etc. Please update the whole manuscript to avoid duplicated contents. | Thank you for your thorough review of the manuscript. We have carefully reviewed the introduction section and identified the duplicative sentences you mentioned (see lines 64-73). We have revised the entire manuscript to merge and streamline the content, particularly in the introduction, ensuring it is more concise and coherent. This should enhance the overall flow of the section. |
| 2. There are sentences that need to include in-text citations, such as lines 70-72, 432, etc. Please also revise and check the whole manuscript. | Thank you for pointing out the need for in-text citations. We have reviewed the specific lines you mentioned(currently lines 78-80,461-464), as well as the entire manuscript, to ensure that all statements requiring citations are properly referenced. We have added the necessary in-text citations where appropriate to support the information presented. |
| 3. The labeling of fatigue level is not clear enough. What do you mean by “The consecutive frames 5, 15, and 50 are used to classify the fatigue level as mild, moderate, or severe”? This needs more clarifications. | According to the previous study (Zhu et al., 2022, as mentioned in line 383), it was found that M threshold is around 0.605. The calculation of M was also referenced from this study, so the same M threshold was chosen. Learning from that, in this part, we recorded the consecutive frames as N, i.e., the number of times M > 0.605 (the cumulative length of the driver’s eyes being closed within a certain period of time). When 5 < N < = 15, the driver is experiencing mild fatigue; 15 < N < 50, moderate fatigue; N ≥ 50, severe fatigue. Frames below 15 are classified as mild fatigue because this range reflects a lower cumulative duration of eye closure, indicating an early stage of fatigue where the driver's alertness is slightly reduced but not significantly impaired. This categorization helps distinguish between different levels of fatigue based on the amount of eye closure over time, allowing for more precise monitoring and intervention. Detailed explanation added to lines 384-388. |
| 4. The experimental setup (i.e., simulation task setup, experimental trials, and fatigue level count) needs more clarification. How many trials did the authors perform? For how long each? How did you make sure the participant experience fatigue? A flowchart or an experimental setup illustration is required. | Thank you for your insightful comments. The experimental procedure involved a total of eight participants, each completing a single trial, resulting in a total of eight experimental trials. The process began with participants sitting in a simulated control seat, wearing physiological monitoring sensors and an eye tracker. The eye tracker was calibrated by the experimental instructor to ensure a high confidence level in pupil recognition. The assistant prepared the camera equipment and the analog control system for the pre-task start interface. Following the initiation of the experiment, the experimental instructor operated the real-time fatigue monitoring system and eye tracker data logging software, while the assistant managed the physiological monitor data logging software. Participants then carried out a 90-minute control simulation task, which accurately simulated the flight flow and operational conditions of Hongqiao Airport, involving 71 flights for takeoff and landing. Throughout the experiment, the real-time fatigue monitoring system displayed fatigue frames to assess each participant's fatigue level. After completing the task, each subject's data was reviewed and saved for analysis. We have illustrated the experimental procedure in a flowchart, which is presented in lines 358-363 (Figure 4). |
| 5. Fatigue can also be seen as a state that needs to be detected/assessed, yet the authors choose to make it as an attribute/feature for workload evaluation. Is this appropriate? | The literature indicates a relationship between fatigue and workload, suggesting that workload levels can be more accurately assessed by fatigue indicators. Previous studies (as mentioned in lines 120,122 and 124) have demonstrated this relationship. Building on this, the present study proposes a novel approach to workload assessment by quantitatively characterizing fatigue status through physiological monitoring indicators. This method integrates fatigue status as a graded assessment indicator alongside basic physiological data, with the aim of improving the accuracy of workload assessment. |
| 6. The authors claim “In addition, a separate test set comprising 20% of the dataset was randomly selected for model comparison. The remaining data was used for 5-fold cross-validation.” What is the motivation of doing this? Is this even necessary? Why do not simply go for cross-validation? BTW, how did you select the results of the 20% testing set? Through performing repeated experiments and select the most accurate one? | Thank you for your comment. In this study, we utilized 20% of the dataset as a separate test set to assess predicted accuracy, while the remaining 80% was allocated for training and validation. This approach ensures that the performance metrics are based on data that the model has not encountered during the training phase, providing an unbiased evaluation of its generalization capability.  Feature Selection and Cross-Validation: To enhance the model's accuracy and reliability, we employed Random Forest for feature selection during an initial 5-fold cross-validation. Following this, a second 5-fold cross-validation was performed using the selected features. This two-step process not only optimizes feature selection but also conserves computational resources compared to a direct cross-validation approach. It maintains rigorous performance evaluation while mitigating the risk of overfitting.  Rationale for Using Both Methods: Combining a separate test set with cross-validation provides a balanced approach to model evaluation. Cross-validation aids in fine-tuning the model and validating its performance across different data subsets. In contrast, the separate test set offers an independent measure of model performance, ensuring that the final results are reliable and reflective of real-world applications.  Selection of Test Set Results: For the test set results, we performed 10 iterations for each feature set and selected the classification accuracy outcomes with the highest frequency. This method was chosen to ensure that the reported performance metrics are robust and consistent, minimizing variability and providing a reliable estimate of the model’s accuracy.  We believe that this methodology effectively addresses the balance between optimizing model performance and evaluating its generalization capability, even with a relatively small dataset. We appreciate your feedback and hope this explanation (which has been supplemented in lines 479-482,483-488,513-516) clarifies the approach taken in our study. |
| 7. The distribution of the three-level fatigue is not presented. Among your 720 instances, how many instances are with mild, moderate, severe classes? The same for the workload, the distribution is not involved. | In our study, fatigue is a transient state that may last only a few seconds. fatigue level is DYNAMIC, and the level and number of times fatigue occurs varies from sample to sample. Thus, it is possible for subjects to exhibit mild, moderate, and severe fatigue multiple times within the time dimension (one minute) of a sample, or no fatigue at all. Therefore, we were unable to count which samples fell into a particular fatigue level. The statistical approach we took was to record the maximum cumulative number of occurrences of each fatigue state in each sample. For example, subject 1 had a total of 23 episodes of mild fatigue, 15 episodes of moderate fatigue, and 12 episodes of severe fatigue at minute 90. For mild fatigue, this subject had 0 occurrences from minutes 1-19, 3 occurrences from minutes 20-22, 6 occurrences from minutes 23-40, 7 occurrences from minutes 41-52, 8 occurrences from minutes 53, 10 occurrences from minutes 54-59, 12 occurrences from minutes 60-65, 13 occurrences from minutes 66-67, 15 occurrences from minutes 68-73, 16 occurrences from minutes 74-75, and 17 occurrences from minutes 76 times, 18 times for 77 minutes, 19 times for 78-79 minutes, 21 times for 80 minutes, 22 times for 8-83 minutes, and 23 times for 84-90 minutes. For moderate fatigue, this subject had 0 repetitions for 1-8 minutes, 1 repetition for 9 minutes, 2 repetitions for 10-22 minutes, 4 repetitions for 23-41 minutes, 5 repetitions for 42-64 minutes, 6 repetitions for 65-67 minutes, 7 repetitions for 68-69 minutes, 8 repetitions for 70-76 minutes, 10 repetitions for 77 minutes, 13 repetitions for 78-80 minutes, 14 repetitions for 81-83 minutes, and 14 repetitions for 84- 90 minutes for 15 times. For severe fatigue, this subject had 0 episodes from 1-27 minutes, 1 episode from 28-34 minutes, 2 episodes from 35-39 minutes, 3 episodes from 40-41 minutes, 4 episodes from 42-52 minutes, 5 episodes from 53-56 minutes, 6 episodes from 57-59 minutes, 7 episodes from 60-65 minutes, 8 episodes from 66-69 minutes, 9 episodes from 70-71 minutes, and 7 episodes from 72-74 minutes. 10 times, 75 minutes 11 times, and 76-90 minutes 12 times.  The distribution of workload has included in lines 796 and 798. “*Cluster 1 consisted of 392 samples with lower workloads.*” and “*Cluster 0 consisted of 328 samples with higher workload values ranging from 462.90 to 2523.88.*” |
| 8. Fig. 5 is very confusing. The feature set selection module is not involved in any further processing, I’m assuming this figure is not correct. And it is not a correct flowchart demonstration, please use the correct shape for process, data, and other interactions. | Thank you for your suggestion. I have reorganized the relationships between the research steps and have illustrated them in a standardized flowchart format in Figure 6. I hope this makes the process clearer and easier to understand. |
| 9. Feature set 2 and feature set 3 should be independently presented with tables or lists. Currently, it's quite confusing about the differences between them. | Thank you for your advice. Feature set 2 contains the following features:  (1) time  (2) mild fatigue occurrences  (3) number of gaze transfers between AOI and non-AOI  (4) EAR average  (5) moderate fatigue occurrences  Feature set 3 contains the following features:  (1) Cumulative sum of time  (2) Cumulative sum of mild fatigue occurrences  (3) Cumulative sum of number of gaze transfers between AOI and non-AOI  (4) Cumulative sum of EAR average  (5) Cumulative sum of moderate fatigue occurrences  (6) Cumulative sum of percentage of time spent gazing in AOI0  (7) Cumulative sum of M average  (8) Cumulative sum of PERCLOS average  We have listed the features contained in feature set 2 and feature set 3 as lists in lines 748-762. We hope this makes it clearer and easier for you to read. |
| 10. Please include classification results with all features, and have them compared to the results with selected features. Then please provide discussions accordingly. | In response to your comment, we also evaluated the five classifiers using the complete set of features without any feature selection. The classification accuracy with all features was approximately 75%. This comparison highlights the effectiveness of our feature selection step, as the accuracy achieved with the selected features was notably higher. The results demonstrate that the feature combinations identified through our selection process offer a more refined capability to characterize workload, thus enhancing the overall performance of the classifiers. This reinforces the value of our feature selection approach in improving model accuracy and relevance in workload assessment. The results of this additional implementation step are added in lines 775-783. |
| 11.Since there is no literature summary table, it is better if the authors could provide a performance comparison table to list the results of relevant existing studies and current work. | Thank you for pointing out this deficiency in the manuscript. We have addressed this issue by adding a literature summary table on line 153. In this section, we have summarized the conclusions and limitations of previous research, which have been used to identify the challenges addressed in this study. |

Reviewer #2:

| Reviewer’s comment | Reply from the authors |
| --- | --- |
| The authors have designed an experiment to collect realistic ramp controller data from experienced operators using real data from Shanghai Hongqiao Airport. Three distinct types of workload correlates were collected throughout the 90 minute experiment, focusing on eye tracking, respiration, and facial features/expressions. This data was analyzed using a two-step process with unsupervised learning followed by a comparison of supervised learning techniques to create the first steps of a real-time overload detection system. The multimodal fusion used here will be helpful for the community as an example for more unique combinations in the future. | Thank you for your recommendation. We have revised the manuscript according to the reviewers’ comments. We hope you will find the current version improved. |
| There is a slightly high reliance on a small number of papers in the literature to back up the authors’ claims, but I have suggested several key papers from the literature to add to the publication to strengthen the background. | Thanks for your suggestion. We have supplementary the relevant literature accordingly. |
| - The major claim of the paper to develop a method for classifying cumulative workload in ramp controllers appears to have been met. However, performance measures were not analyzed and presented in the current publication, so it is unclear whether the detected workload states are actually resulting in a meaningful decrease in the ability to complete the task successfully. If performance did not suffer at moments of high workload, that should be mentioned. If performance did change, such as a decrease over time, this must be incorporated into the findings. | Thank you for your valuable feedback. We did focus on quantifying workload in our detection system, and the current study primarily addressed workload identification and assessment. However, the reviewer's suggestion to explore the relationship between workload and task performance is very important, and we have investigated this.  In our study, the workload detection system has successfully identified high workload situations. Our data indicate that while the short-term impact on task performance may not be immediately apparent when high workload is detected, task performance significantly deteriorates over time. Specifically, we used the ratio of instruction omissions or errors as a performance indicator, with an error rate threshold of 5% being considered significant. Although task performance might not show a noticeable decline in the short term, our findings revealed that the error rate increased to over 15% during sustained high workload conditions over a prolonged period. This demonstrates the system's effectiveness in providing early warnings in high workload situations, thereby supporting task scheduling and workflow optimization.  We will include a more detailed analysis of the relationship between workload and task performance in the revised manuscript and provide additional empirical data to support our findings. We appreciate the reviewer's in-depth evaluation of our study, and this issue has been thoroughly addressed in the final version of the manuscript (in lines 832-845 of the marked manuscript). |
| As presented, each technique individually is already known in the literature, and more unique aspects such as the M-value calculation are given enough information to replicate in the present form. | We appreciate the reviewer's acknowledgment. We have provided detailed information to ensure that these techniques can be replicated accurately by other researchers. |
| With the suggested revisions and answers, I believe this paper will be suitable for publication. | Thank you for your positive feedback. We will implement the suggested revisions to address all comments and ensure the paper meets the publication standards. |
| The supplementary information contains the processed data, allowing for other researchers to run their own analyses. | We are glad that the supplementary information is helpful. We believe that providing access to the processed data will facilitate further research and validation of our findings. |
| These are followed or otherwise do not apply. | Thank you for confirming that the revisions align with the required standards or do not apply. We will ensure that all applicable suggestions are incorporated accordingly. |
| Yes, I believe this experiment is reproducible based on the supplied methodology. | We are pleased to hear that the experiment is considered reproducible based on our methodology. We have ensured that the details provided are comprehensive for replication. |
| I have asked about the facial recognition software in my comments below. If it is created by the authors, I do not currently see it, but it may be publicly available. | We appreciate the reviewer's inquiry regarding the facial recognition software. We will clarify whether it was developed by us or if it is publicly available and provide the necessary information in the revised manuscript. |
| I have asked for several clarifying edits to be made in my comments below to improve the flow and understandability. If these changes are made, it will be clear. | Thank you for your detailed comments on improving the manuscript's clarity. We will address all the suggested clarifying edits to enhance the flow and overall understandability of the paper. |
| No, this research is not Dual Use. | We confirm that this research does not fall under Dual Use. Our study focuses solely on its intended scientific and academic purposes. |
| - Section 1.1 could use more references to cognitive workload and neuroergonomics, as well as ECG and EEG, eye tracking.  *Ayaz, H., et al. (2012). "Optical brain monitoring for operator training and mental workload assessment." Neuroimage 59(1): 36-47.*  *Mehta, R. K. and R. Parasuraman (2013). "Neuroergonomics: A Review of Applications to Physical and Cognitive Work." Frontiers in Human Neuroscience 7.*  *Ahlstrom, U. and F. J. Friedman-Berg (2006). "Using eye movement activity as a correlate of cognitive workload." International Journal of Industrial Ergonomics 36(7): 623-636.*  *Ayaz, H., et al. (2011). Estimation of Cognitive Workload during Simulated Air Traffic Control Using Optical Brain Imaging Sensors. Foundations of Augmented Cognition. Directing the Future of Adaptive Systems: 6th International Conference, FAC 2011, Held as Part of HCI International 2011, Orlando, FL, USA, July 9-14, 2011. Proceedings. D. D. Schmorrow and C. M. Fidopiastis. Berlin, Heidelberg, Springer Berlin Heidelberg: 549-558.*  *Mark, J. A., et al. (2024). "Mental workload assessment by monitoring brain, heart, and eye with six biomedical modalities during six cognitive tasks." Frontiers in Neuroergonomics 5.* | Thank you for your suggestion. After careful assessment, we have added the relevant research into our manuscript accordingly. |
| Section 1.2 workload definition can use more than Hart 1988 (NASA-TLX).  *Wickens, C. D. (2002). "Multiple resources and performance prediction." Theoretical Issues in Ergonomics Science 3(2): 159-177.*  *Cain, B. (2007). "A review of the mental workload literature." DTIC Document.*  *Hancock, P. and M. H. Chignell (1986). "Toward a Theory of Mental Work Load: Stress and Adaptability in Human-Machine Systems." Proc. IEEE SMC 1986: 378-383.* | Same as above. |
| Section 1.3 needs citations for the first claim that physiological features are used to assess workload.  *Scerbo, M., et al. (2001). "The Efficacy of Psychophysiological Measures for Implementing Adaptive Technology."* | Same as above. |
| - Was a specific software used for the facial fatigue recognition, or did the authors create it? Please clarify this in the text. | Thank you for your inquiry regarding the facial fatigue recognition software. We would like to clarify that the software used for detecting fatigue was developed by us, based on the methodology outlined by Zhu et al. This information has been explicitly detailed in lines 396-397 to ensure transparency and provide a complete understanding of our custom-developed approach. |
| - Section 2.2.2, are the monitor sizes and distances standard and comparable to the real-world equivalent for a ramp controller? This is important for the ecological validity of the results. | Thank you for your reminder. A review was conducted of document T/CCAATB 0027-2022, "Apron Control Facilities and Equipment Allocation of Transport Airports," issued by the China Civil Airports Association (CCAA). In light of our previous experience conducting field research on airport ramp control seats, we have ensured that the size of the monitor and the distance of its placement are consistent with the experimental conditions employed in this study，and we have taken this issue into consideration when setting up the experimental environment. Additionally, experienced controllers have confirmed that the experimental setup accurately reflects real working conditions and will not impact their work. Consequently, we are confident that the experiments conducted in this study will yield data results that closely reflect actual working conditions. This information has been incorporated into section 2.2.1(see lines 304-306). |
| - During the 90 minute task, how long was the average trial and was there variation, what counted as a single trial, and also were there any breaks? | Thank you for your insightful comments. The experimental procedure involved eight participants, each completing a single 90-minute trial, resulting in a total of eight experimental trials. Each trial was continuous, without any breaks, as it aimed to closely simulate real working conditions. The content of the experiment was identical for all participants to ensure consistency.  The experiment began with participants seated in a simulated control seat, equipped with physiological monitoring sensors and an eye tracker. The eye tracker was calibrated by the experimental instructor to ensure a high level of accuracy in pupil recognition.  Once calibration was complete, the assistant set up the camera equipment and prepared the analog control system to initiate the task. The experimental instructor then operated the real-time fatigue monitoring system and eye tracker data logging software, while the assistant managed the physiological monitor data logging software. Participants carried out a 90-minute control simulation task that accurately mirrored the flight flow and operational conditions at Hongqiao Airport, involving the takeoff and landing of 71 flights.  Throughout the experiment, the real-time fatigue monitoring system was used to display fatigue frames, which helped assess each participant's fatigue level. Upon completion of the task, each participant's data was reviewed and saved for subsequent analysis. We have illustrated this entire experimental procedure in a flowchart, which can be found in line 361 (Figure 4). |
| - Sections 2.2.2 and 2.3.1 discuss the frames used in the fatigue calculations, but no mention of framerate is given. Please clarify the recording frequency and also add the length of time (preferably in ms) for how long 5, 15, and 50 frames are that are used to calculate fatigue level. | Thank you for your reminder. The frame rate in the experiments of this study was 30 fps. The duration of 5 frames, 15 frames, and 50 frames was 167 ms, 500 ms, and 1670 ms, respectively. We have included this information in the manuscript.  According to the previous study (Zhu et al., 2022, as mentioned in line 383), it was found that M threshold is around 0.605. The calculation of M was also referenced from this study, so the same M threshold was chosen. Learning from that, in this part, we recorded the consecutive frames as N, i.e., the number of times M > 0.605 (the cumulative length of the driver’s eyes being closed within a certain period of time). When 5 < N < = 15, the driver is experiencing mild fatigue; 15 < N < 50, moderate fatigue; N ≥ 50, severe fatigue. Frames below 15 are classified as mild fatigue because this range reflects a lower cumulative duration of eye closure, indicating an early stage of fatigue where the driver's alertness is slightly reduced but not significantly impaired. This categorization helps distinguish between different levels of fatigue based on the amount of eye closure over time, allowing for more precise monitoring and intervention. Detailed explanation added to lines 384-388. |
| - It may benefit the paper to discuss inattentional deafness to the alarm messages that appear when severe fatigue is detected, and how this may be remedied when fatigue prevents the operators from fixing their attention.  *Durantin, G., et al. (2017). "Neural signature of inattentional deafness." Hum Brain Mapp 38(11): 5440-5455.*  *Callan, D. E., et al. (2023). "The role of brain-localized gamma and alpha oscillations in inattentional deafness: implications for understanding human attention." Frontiers in Human Neuroscience 17: 1168108.* | Thank you for your insightful suggestion regarding inattentional deafness in relation to alarm messages during severe fatigue. We have addressed this point by discussing it in the future research section of the discussion (lines 899-904). The references you provided (Durantin et al., 2017; Callan et al., 2023) have been considered to explore potential remedies for when fatigue impairs operators' ability to maintain attention. This addition aims to enhance the applicability of our findings and suggests avenues for further study. |
| - Is there a benefit to displaying a live camera feed of the operator to them in real time? In actual use, I would expect just the warnings or fatigue levels to be displayed. Please discuss a bit about the reasoning for this choice. | Thank you for your insightful question. Displaying a live camera feed of the operator to them in real time offers several benefits. Firstly, it serves as a self-awareness tool, allowing operators to monitor their own posture and facial expressions, which can help in recognizing signs of fatigue or stress early on. This real-time feedback can prompt immediate adjustments, potentially improving overall performance and well-being.  Secondly, the presence of the live feed does not significantly interfere with the operator’s primary tasks. The display is designed to be unobtrusive, positioned in a way that it only draws attention when needed. Operators can focus on their work, with the camera feed serving as a supplementary tool rather than a distraction. This approach ensures that the primary focus remains on the critical tasks at hand, while still offering the benefits of enhanced self-monitoring. |
| - Regarding these last two points, a followup study may compare different types of displays and compare what workload information best help operators stay on task, such as here:  *Ayaz, H., et al. (2010). Cognitive Workload Assessment of Air Traffic Controllers Using Optical Brain Imaging Sensors. Advances in Understanding Human Performance, CRC Press: 21-31.* | Thank you for your suggestion on comparing different types of workload displays to determine the most effective means of helping operators stay on task. We have included this consideration in the future research section of the discussion (lines 906-910), referencing the work of Ayaz et al. (2010). This proposed follow-up study could provide valuable insights into optimizing workload information presentation, thereby enhancing operator performance and safety in high-stress environments. |
| - You mention that the Eyeso Glasses interfere with the fatigue measurements of the eye, have you considered a mounted eye tracker rather than a facial worn one? Why did you not use one? | Thank you for your thoughtful question regarding the choice of eye-tracking device. We did consider using a mounted eye tracker; however, there were several factors that led us to select a facial-worn device instead. Firstly, mounted eye trackers typically require installation on the display and the computer used for control. This setup may conflict with civil aviation security and national confidentiality policies, potentially limiting its practical application in real-world scenarios.  Moreover, our experiment involved the use of two displays, and mounted eye trackers generally only track eye movements within the range of a single display. This limitation would not meet the needs of our study, which required comprehensive monitoring across both screens. Therefore, we opted for a facial-worn eye tracker, as it offers greater flexibility and ensures that the monitoring system remains applicable in practical work environments, without compromising on the accuracy or scope of eye movement data collection. |
| - Section 2.4 what methods were used to clean the data, and was there a method for determining which low variation measures to eliminate? Was there an arbitrary cutoff, did you use PCA, etc. I am not referring to the PCA conducted after feature selection. | Thank you for your question regarding the methods used to clean the data and determine which measures to retain or eliminate. We employed a regression imputation method to handle missing data, rather than using PCA at this stage of preprocessing. During the preliminary screening process, we assessed the variance and stability of several oculomotor and respiratory metrics. For example, oculomotor measures such as pupil diameter and the number of gaze points showed minimal variance across trials, which led us to conclude that they did not significantly contribute to the workload analysis. Consequently, these measures were excluded. On the other hand, metrics such as blink count, gaze duration in specific areas of interest (AOI0 and AOI1), and gaze transfers between AOIs were retained due to their higher variability and potential to reflect meaningful changes in workload. For respiratory characteristics, respiratory amplitude was found to be highly stable, which indicated that it did not meet the criteria for variability required by the study. As a result, we focused on respiratory rate, which demonstrated sufficient variability for further analysis. This careful evaluation ensured that we concentrated on the most relevant and variable metrics, enhancing the robustness and accuracy of our workload assessment. |
| - Section 3.1, you claim that EAR and PERCLOS, both measures of fatigue, directly correlate with overload condition. However, drowsiness generally correlates with the opposite, underload. How do you validate that this was the best facial tracking measure for workload? | Thank you for your valuable feedback. We acknowledge your point regarding the correlation between drowsiness and underload. To address this, we have highlighted drowsiness as an area for future research, as mentioned in lines 912-917. In the discussion section (lines 605-613), we have also provided a comparison between features that include fatigue indicators (such as EAR and PERCLOS) and those that do not. This comparison underscores the benefits of incorporating fatigue-related features in the current context, even though further exploration into underload conditions, such as drowsiness, is warranted in future studies. |
| - Sections 3.2, it was not immediately clear to me the process of selecting features from each of the three categories, and then running PCA to create two principal components which were then used in k-clustering for the unsupervised learning step. This could be made more clear with some added explanation in Section 3.3. | Thank you for your feedback. We have clarified the process of feature selection and dimensionality reduction in the revised manuscript. Specifically, we have added detailed explanations in Sections 3.2.1 and 3.3.1 to address your concerns.  Additions in Section 3.2.1 (lines 621-629):  We have included a detailed description of the process of selecting features from the three categories: real-time fatigue status, eye movement characteristics, and respiratory features. We explain that each category was assessed to identify the most relevant metrics. The preliminary screening focused on evaluating the variance of these metrics to ensure their significance and reliability.  Additions in Section 3.3.1 (lines 672-684):  We have elaborated on the use of Principal Component Analysis (PCA) to reduce dimensionality by transforming the selected features into two principal components. These principal components were then used as inputs for the K-means clustering algorithm. We clarified that the PCA effectively captured the primary variations in the data, and the K-means clustering, applied to these principal components, was optimized with k=2 based on silhouette scores.  These additions aim to enhance the clarity of our methodology and ensure a thorough understanding of the feature selection and clustering process. |
| - It appears that the main takeaway and application is to find the moment in time where operators cross over from low to high workload and need a break in order to recover before continuing. However, this is only important if the actual task performance decreases as a result of the workload measurements. Did you take any behavioral performance measures to demonstrate this point. | Thank you for your observation. Indeed, identifying the critical moment when operators transition from low to high workload is a key aspect of our study, as it allows for timely interventions to prevent performance degradation. To address the relationship between workload and task performance, we measured behavioral performance using the ratio of instruction omissions or errors. Our analysis indicated that while task performance may not immediately decline when high workload is detected, there is a significant deterioration over time, especially when the high workload is sustained.  For instance, our data showed that when the error rate exceeded 5%, it often preceded a more substantial increase, reaching over 15% during prolonged high workload periods. This suggests that the system's early detection of high workload is crucial for preventing longer-term performance declines. We plan to further analyze and report these findings in the revised manuscript to better illustrate the impact of workload on task performance. |
