# Supplementary material for: A numerical evaluation of real-time workloads for ramp controller through optimization of multi-type feature combinations derived from eye tracker, respiratory, and fatigue patterns
Source: PLoS One. 2024 Nov 8;19(11):e0313565. doi: 10.1371/journal.pone.0313565 (PMC11548742; doi:10.1371/journal.pone.0313565)
Supplement: Attachment — Submitted filename: Response to Reviewers-R2.docx [file pone.0313565.s003.docx]

**Response to reviewers on the paper entitled “A numerical evaluation of real-time workloads for ramp controller through optimization of multi-type feature combinations derived from eye tracker, respiratory, and fatigue patterns”**

*Manuscript ID:* *PONE-D-24-23960*

*Journal: PLOS ONE*

*Revision: R2*

We would like to express our gratitude and appreciation to the reviewers for providing valuable comments to ensure our manuscript’s continuing improvement. All the comments provided by the different reviewers have been acted upon to improve the manuscript. We hope that the editors and reviewers find this revision an improvement.

In accordance with the comments from the editors and reviewers, amendments are made as given below:

Journal Requirements &Editor:

| Journal Requirements & Editor’s comment | Reply from the authors |
| --- | --- |
| Please review your reference list to ensure that it is complete and correct. If you have cited papers that have been retracted, please include the rationale for doing so in the manuscript text, or remove these references and replace them with relevant current references. Any changes to the reference list should be mentioned in the rebuttal letter that accompanies your revised manuscript. If you need to cite a retracted article, indicate the article’s retracted status in the References list and also include a citation and full reference for the retraction notice. | Thank you for your helpful reminder. We have carefully reviewed and updated the reference list to ensure it is complete and accurate. In addition, we have checked for any retracted papers and can confirm that none have been cited. To further improve the accuracy of our references, we used the EndNote tool provided on the journal's official website (https://endnote.com/style_download/plos-public-library-of-science-all-journals/). The updated reference list is now reflected in the revised manuscript. We appreciate your guidance and attention to this matter. |
| Follow the formatting guidelines and proofread for acceptance as requested by a reviewer. | Thank you for the reminder. We have carefully followed the formatting guidelines provided by the journal and thoroughly proofread the manuscript to ensure compliance. The necessary adjustments have been made to meet the requirements for acceptance, as suggested by the reviewer. |

Reviewer #1:

| Reviewer’s comment | Reply from the authors |
| --- | --- |
| Thanks to the authors for putting effort in revising this manuscript, I find the revised version more solid and clear. And I'm happy with most of their responses. | Thank you for your positive feedback and for acknowledging the improvements in the revised manuscript. We appreciate your thoughtful comments and are glad that the revisions have strengthened the clarity and overall quality of the work. If there are any further suggestions, we are happy to address them. Thank you again for your time and valuable insights. |
| 1. Please correct the in-text citation styles. For example, in line 68, it should be "He et al. [6]" rather than "[6]". Please check the entire manuscript. | Thank you for your valuable feedback. We have corrected the in-text citation styles as per your suggestion. Specifically, we have revised the citations in lines 65, 887, and 894 of the “Revised Manuscript with Track Changes-R2” document to ensure proper formatting. We appreciate your attention to detail and have carefully checked the entire manuscript for consistency. |
| 2. When use in-text citations, there should be a space after the sentence. For example, in line 77, it should be "... the cardiac cycle [8-10]" and "... correlating workload states [11,12]" in line 80. Please check the entire manuscript. | Thank you for your careful review and helpful comments. We have addressed the spacing issue after in-text citations as you suggested. Specifically, we made the necessary corrections in lines 74, 77, 410, 452, and 488 of the “Revised Manuscript with Track Changes-R2” document. We appreciate your attention to detail and have thoroughly checked the entire manuscript to ensure consistency. |
| 3. For existing literature comparison (Table 1), it is expected to list the methodology peformance. | Thank you for your valuable feedback. We have revised Table 1 to include the methodology performance in the original methodology description column, as you suggested. This update provides a clearer comparison of the methodologies used in the existing literature. We appreciate your guidance in improving the quality of the manuscript. |
| 4. The authors should carefuly go through proofreading before the paper gets accepted. There are many typo mistakes that need to be revised. Hope this will help the authors to form into a more acceptable version. | Thank you for your constructive feedback. We apologize for any typographical errors present in the manuscript. We have thoroughly proofread the entire document to identify and correct all typos and mistakes to enhance the clarity and quality of the paper. We appreciate your attention to detail and your commitment to improving the manuscript. |

Reviewer #2:

| Reviewer’s comment | Reply from the authors |
| --- | --- |
| All comments have been addressed. | Thank you for your positive feedback. We are glad to hear that all comments have been addressed to your satisfaction. If you have any further suggestions or questions, please feel free to let us know. We appreciate your time and effort in reviewing our manuscript. |
